# Combined effects of photorespiration and fire strongly regulate atmospheric oxygen levels

**DOI:** 10.1126/sciadv.ady0542

**Published:** 2026-01-07

**Authors:** Rayanne Vitali, Claire M. Belcher, Benjamin J.W. Mills, Andrew J. Watson

**Affiliations:** ^1^ Department of Geography, University of Exeter, Prince of Wales Road, Exeter EX4 4PS, UK.; ^2^ Department of Environmental Science, Aarhus University, Frederiksborgvej 399. DK-4000 Roskilde, Denmark.; ^3^ School of Earth and Environment, University of Leeds, Leeds LS2 9JT, UK.

## Abstract

Atmospheric oxygen concentrations have remained remarkably stable over the past ~400 million years (Myr), suggesting the presence of robust regulatory mechanisms. Because of its sensitivity to oxygen, wildfire was traditionally assumed to control oxygen levels by limiting terrestrial vegetation; however, this feedback is nullified by high moisture levels in tropical ecosystems. Using vegetation modeling, we show that where oxygen-fire effects are dampened by high moisture, photorespiration becomes more effective through increased temperatures. Together, these processes interact to drive an 86% reduction in modeled global biomass when oxygen levels reach 35%. This coregulation imposes substantially tighter control of atmospheric oxygen than wildfire alone, providing previously unknown insights into the spatial and interactive feedbacks that may explain the remarkable stability of oxygen levels since the evolution of forests.

## Teaser

Interactions between wildfire and photorespiration under high O_2_ strongly regulate Earth’s atmospheric oxygen levels.

## INTRODUCTION

Since the establishment of forest ecosystems ~420 million years ago (Ma), atmospheric oxygen (O_2_) has remained at broadly present atmospheric levels (PALs) (*1*), estimated to have remained between the range ~15 to 40 vol % O_2_ (*2*), playing a vital role in the evolution of life on Earth (*3*, *4*). This remarkably small variation in atmospheric O_2_, despite the whole atmospheric inventory being completely replaced more than 100 times throughout this period, has been termed “the oxygen puzzle” (*1*). The stability of oxygen over such a long period suggests that regulatory mechanisms must be in place to prevent oxygen rising or falling out of bounds. Land-based solutions, particularly fire-feedbacks on oxygen, have been prevalent in explaining the regulation of atmospheric O_2_ through time (*1*, *3*, *5*–*8*). Wildfire can regulate atmospheric O_2_ concentration through changes to organic carbon burial; the main source of atmospheric O_2_ over geological timescales (fig. S1A) (*1*, *5*, *9*). Studies have found that the probability of ignition and rate of spread of a fire increases sharply with rising oxygen levels (*10*–*13*). Therefore, fire is able to act as a negative feedback on atmospheric O_2_ through suppressing terrestrial vegetation productivity and subsequently the amount of organic carbon burial that can occur when oxygen levels are high and vice versa (*1*, *5*).

Despite decades of work on this problem, the supposed tight regulation of oxygen levels has remained largely untested and the physical upper limit of atmospheric O_2_ on Earth has been long disputed. The long-standing assumption has been that atmospheric O_2_ levels greater than ~35 vol % O_2_ would threaten the destruction of global forests by fire due to enhanced fire frequency, extreme fire behavior, and short fire return intervals (*1*, *10*, *11*, *14*), effectively setting an upper limit for atmospheric O_2_. However, dynamic vegetation modeling under changing O_2_ levels questions the strength of the fire feedback at the global scale, showing that the effect of fire on global vegetation under rising oxygen should be mediated by high moisture levels in productive tropical forest ecosystems (*14*). This highlights the need to revisit established assumptions and explore additional feedbacks and regulatory mechanisms. Modern modeling frameworks, including dynamic vegetation models, provide valuable tools to test these processes but require further development to capture more complete biogeochemical interactions relevant to long-term oxygen stability.

Here, we expand O_2_-sensitive vegetation modeling to consider important coregulation effects that can influence carbon burial through variations in plant productivity. In C3 plants, the ribulose-1,5-bisphosphate carboxylase/oxygenase (Rubisco) enzyme has maintained a dual capacity to bind to either CO_2_ or O_2_. Increasing the oxygen mixing ratio causes an inhibitory effect on photosynthetic CO_2_ fixation and increases photorespiration, resulting in lower productivity, a phenomenon often referred to as the Warburg effect (*9*, *15*–*18*) (see fig. S1B). This direct mechanism has been approximated as part of global oxygen feedbacks in simple biogeochemical box models by introducing an arbitrary reduction in terrestrial productivity when O_2_ is high (*19*), but its strength and relationship to other local environmental and physiological parameters have not been established, and thus, the global effectiveness of photorespiration as an oxygen regulation mechanism is not known.

Various studies have investigated the changes in photorespiration and productivity in C3 plants under varying CO_2_ and O_2_ concentrations (*16*–*18*, *20*) and have led to the classification and development of compensation points. The CO_2_ compensation point (γ_c_*) is defined to be the CO_2_ concentration at which net CO_2_ fixation is zero at a given temperature and O_2_ concentration (*16*, *17*, *21*). A less explored concept is the O_2_ compensation point (γ_o_*), which is the O_2_ concentration at which photosynthesis and the opposite respiratory processes are in equilibrium for a given temperature and CO_2_ concentration (*18*). Allowing for variations in both CO_2_ and O_2_ concentrations, we can therefore produce a compensation line that determines the overall photorespiration effect on productivity (*16*, *18*). From this, we can then define a function that describes how altering the CO_2_:O_2_ ratio for a given temperature changes the net photosynthesis of C3 vegetation and hence affects growth, total biomass, and subsequently carbon burial.

To test the impact of varying oxygen levels on the abundance and distribution of global vegetation, we use a version of the Lund-Potsdam-Jena (LPJ)–LMfire Dynamic Global Vegetation Model (DGVM) (*22*) to include oxygen-fire and oxygen-photorespiration effects on terrestrial biomass. Within the model, we simulate oxygen effects on fire through inclusion of relationships between probability of ignition, moisture of extinction, and heat of combustion with atmospheric O_2_ following the approach of Vitali *et al.* (*14*). We then include, as a single function, updated CO_2_:O_2_ compensation points defined by André (*18*) for photorespiration. While previous authors have suggested both photorespiration (*15*–*18*) and fire (*1*, *9*) could have a role in oxygen regulation, this study is this first to include both mechanisms alone and as a combined impact at the global scale using a dynamic vegetation model. This combined and spatially resolved approach is critical because there are likely strong interactions between both effects, for example, photorespiration will affect the amount of fuel (vegetation biomass) available for wildfires. We first run a series of simulations for the present-day climate but excluding human influence over a range of possible atmospheric O_2_ concentrations. We then repeat these simulations at both high CO_2_ and increased temperature as has been the case during many periods in Earth history but using modern day continental configuration. To understand the importance of fire and photorespiration effects on long-term oxygen regulation, we also implement the results from the LPJ-LMfire simulations for fire and photorespiration effects on global vegetation into the Carbon-Oxygen-Phosphorus-Sulphur-Evolution (COPSE) global biogeochemical model (see Materials and Methods). We analyze the impacts of individual and combined feedbacks on atmospheric O_2_ over geological timescales.

## RESULTS AND DISCUSSION

### Oxygen-driven effects on vegetation via fire and photorespiration.

**Fig. 1.**
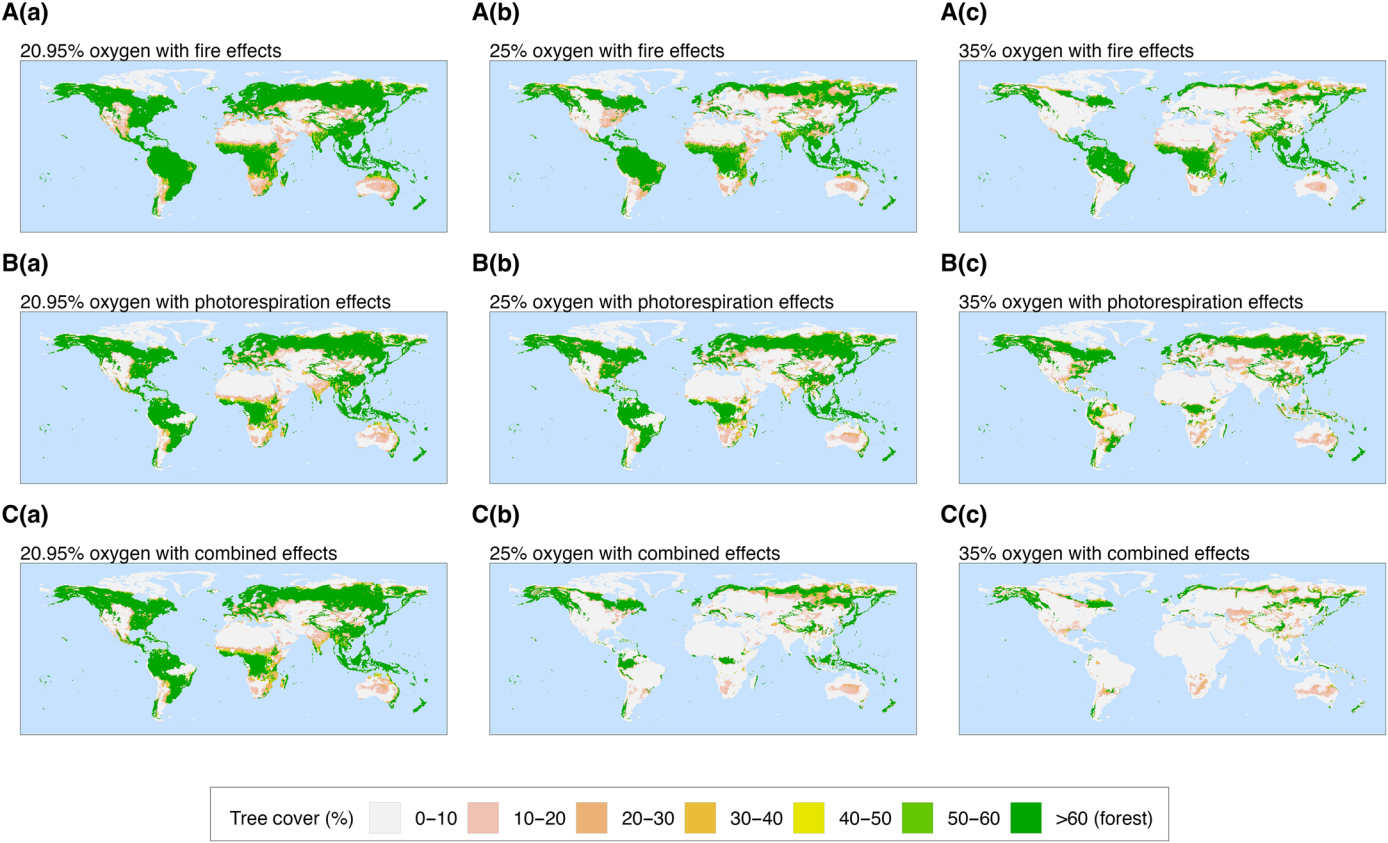
Plots of global tree cover (%) from oxygen simulations. (**A**) Oxygen-fire effects only, (**B**) oxygen-photorespiration effects only, and (**C**) both oxygen-fire and oxygen-photorespiration effects, and output is plotted for (a) 20.95 vol % O_2_ (PAL), (b) 25 vol % O_2_ and (c) 35 vol % O_2_. Plots are taken as 10-year annual averages with forest cover defined to be tree cover greater than 60%.

**Fig. 2.**
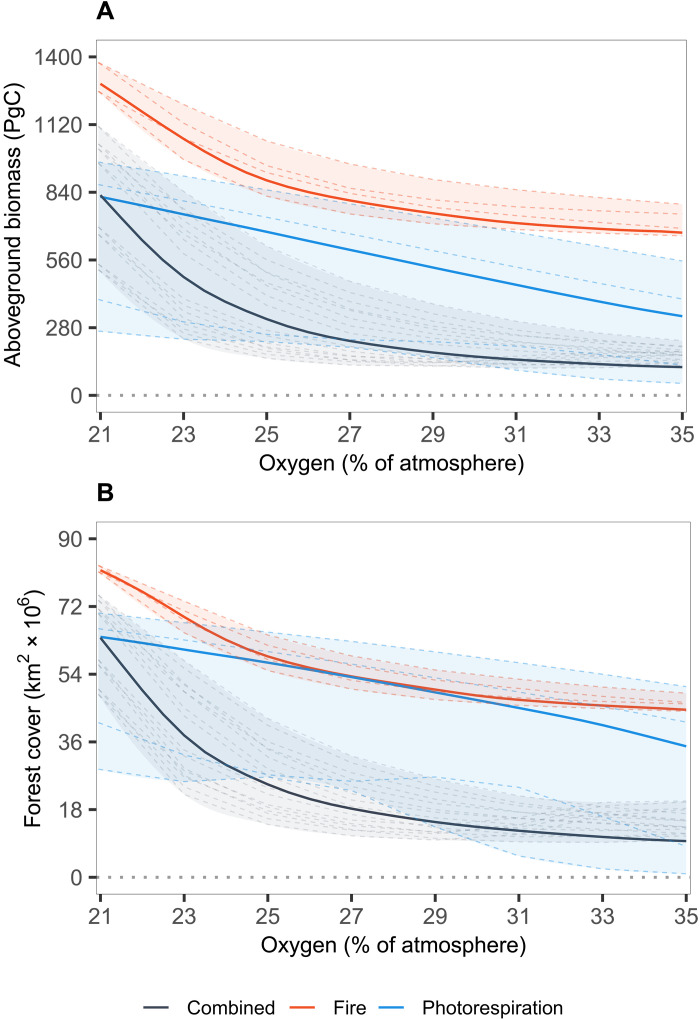
Plots of global total vegetation from LPJ-LMfire simulations over atmospheric oxygen. (**A**) Aboveground biomass (PgC) and (**B**) forest cover (tree cover > 60%, km^2^). Lines indicate different oxygen simulations, which include oxygen-fire effects only (red), oxygen-photosynthesis effects only (blue), and both oxygen-fire and oxygen-photosynthesis effects (black). Shaded areas and dashed lines indicate the range and results from the sensitivity analysis (see Materials and Methods and Supplementary Methods). Totals are calculated from 10-year annual averages from LPJ-LMfire output, summed over grid cells to give a single global value.

Simulations including oxygen effects on fire alone (Fig. 1A, a to c) replicate those conducted by Vitali *et al.* (*14*) and show a decrease in mid- and high-latitude forest cover under increasing atmospheric O_2_, reducing global forest cover and biomass by ~45% at 35 vol % O_2_ compared to PAL (Fig. 2, red lines). Although the effects on tree and forest cover are notable, the sensitivity of forests to increased fire under high-atmospheric O_2_ was shown in that study to be less than had previously been assumed based on laboratory experiments (*1*, *14*, *23*). Previous estimates for the “fire window’ upper limit of atmospheric O_2_ through time have suggested that levels of 25 to 35% O_2_ would threaten the regeneration of present-day forests globally (*1*, *9*, *11*, *13*, *24*, *25*). Yet, the simulations suggest that climatic limitations on fire result in substantial forest cover persisting even at 35 vol % O_2_. Vitali *et al.* proposed that although the number of fires that ignite under high oxygen increase sharply, the rate of fire spread is limited in regions where fuel moisture content remains high (*14*), explaining why forest cover remains at low latitudes where rainforests stay wet and humid year-round and at high latitudes where low temperatures and reduced evaporative demand retain high fuel moisture contents. Hence, oxygen-fire effects on vegetation were indicated to be weaker than inferred from controlled burning experiments.

Alternatively in Fig. 1B (a to c), we do not consider fire but instead apply photorespiration effects in the model for changing O_2_ levels. We find that simulations including this effect on plant productivity alone also suggest that ~45% of forest cover is suppressed at 35 vol % O_2_ (Fig. 2B, blue line). This is comparable to experimental studies such as Beerling *et al.* (*20*), which found that photosynthetic rates halved under 35% O_2_ (*20*, *26*). While fire alone and photorespiration alone appear to reduce global forest cover by roughly the same amount, each influences different areas of the globe. Oxygen-fire effects favor removing mid-high latitudinal forests while oxygen-productivity effects remove forests mainly from low-mid latitudes under increasing oxygen levels (Fig. 1B, a to c). While the former is due to moisture content, the latter is due to the latitudinal temperature gradient. Low latitudes experience higher temperatures, causing the solubility of CO_2_ to decrease and altering the specificity factor of rubisco. This results in an increase of the compensation point for a given O_2_ concentration (*15*, *27*–*29*). In simulations under high atmospheric oxygen, this means warmer temperatures at low latitudes experience more pronounced oxygen inhibition on productivity, while the oxygen-photorespiration effect is more diminishing at higher latitudes with cooler temperatures, reflecting the change in forest cover in these regions compared to PAL [see Fig. 1B(c)].

Fire has been assumed to have a greater sensitivity to changes in oxygen concentration through time than the effect on plant productivity through photorespiration (*1*, *9*). It has been suggested that the present day suppression due to photorespiration is ~30% (*30*). Early DGVM simulations conducted by Bond *et al.* (*23*) found under PAL, fire suppressed forest cover by 50%, implying that fire is more sensitive than oxygen-productivity effects. These findings have strongly influenced the development of global biogeochemical models that predict the abundance of atmospheric oxygen and carbon dioxide levels over geological time (*1*, *31*, *32*). However, we show here that not only are both oxygen-fire and oxygen-plant productivity effects on global vegetation likely to be broadly similar but are also often additive in nature due to their spatial patterns of effect.

Oxygen-fire relationships are critically dependent on fuel moisture, and oxygen-photorespirations feedbacks are dependent on temperature, which means that each feedback alone has an impact on different regions of the globe. In simulations that combined the oxygen effects on both fire and photorespiration (Fig. 1C, a to c), a much larger decrease in total biomass is observed compared to imposing either of the feedbacks individually. Here, at 35 vol % O_2_, forest cover and biomass see a ~86% reduction compared to PAL (Fig. 2, black lines). A substantial amount of forest cover is removed under high oxygen concentrations due to the additive effects of both oxygen-fire feedbacks that remove mainly the mid-high-latitude tree cover and oxygen-productivity feedbacks that remove forest cover from mainly low latitudes. Moreover, the enhanced removal of biomass occurs when including both effects due to the complex interactions between fire and productivity. As oxygen levels rise and cause a greater inhibitory effect on plants, plant growth and hence tree heights become more limited (see fig. S3). This results in a higher chance of mortality if fires occur, which becomes increasingly more likely under rising oxygen. These results indicate that the interaction between fire and plant productivity under high oxygen concentrations has a very substantial effect on the abundance of global vegetation, the rate of organic carbon burial, and the ultimate source of oxygen to the atmosphere over geological time (*1*, *5*). We note that while evapotranspiration is internally calculated in the model as part of vegetation-climate interactions, the role of evapotranspiration-driven water recycling in facilitating forest expansion or enhancing forest resilience in arid regions is not explicitly assessed in this study.

Simulations assessing the sensitivity of parameterizations for fire and photorespiration (see supplementary methods) show reasonable agreement with the results presented above (dashed lines, Fig. 2). Among the parameters tested, those related to photorespiration demonstrated the highest sensitivity, as indicated by the broadest range of outcomes when simulations were conducted using the extrema. This heightened sensitivity stems from the wide range of plausible values for dark respiration rates, which, can vary between 5% and 30% of total oxygen evolution (*33*, *34*). The upper end of this range, characterized by elevated dark respiration rates, amplifies the effects of photorespiration, leading to a notable reduction in biomass and forest cover under PAL. In the primary series of O_2_ simulations presented (Fig. 2, solid lines), dark respiration was assumed to be 10% of total O_2_ evolution, a value situated at the lower end of the plausible range. Consequently, the selected parameters for dark respiration and, by extension, photorespiration yield a relatively modest compensation point, resulting in a lower sensitivity to varying O_2_ concentrations. Higher parameterization values would therefore exacerbate the impact of photorespiration under increasing O_2_ concentrations. Conversely, simulations focusing on fire, as well as those incorporating combined parameterizations, displayed minimal variation even when different combinations of extrema were applied. The combined simulations showed only slightly greater variability, attributable to the sensitivity of photorespiration as discussed.

**Fig. 3.**
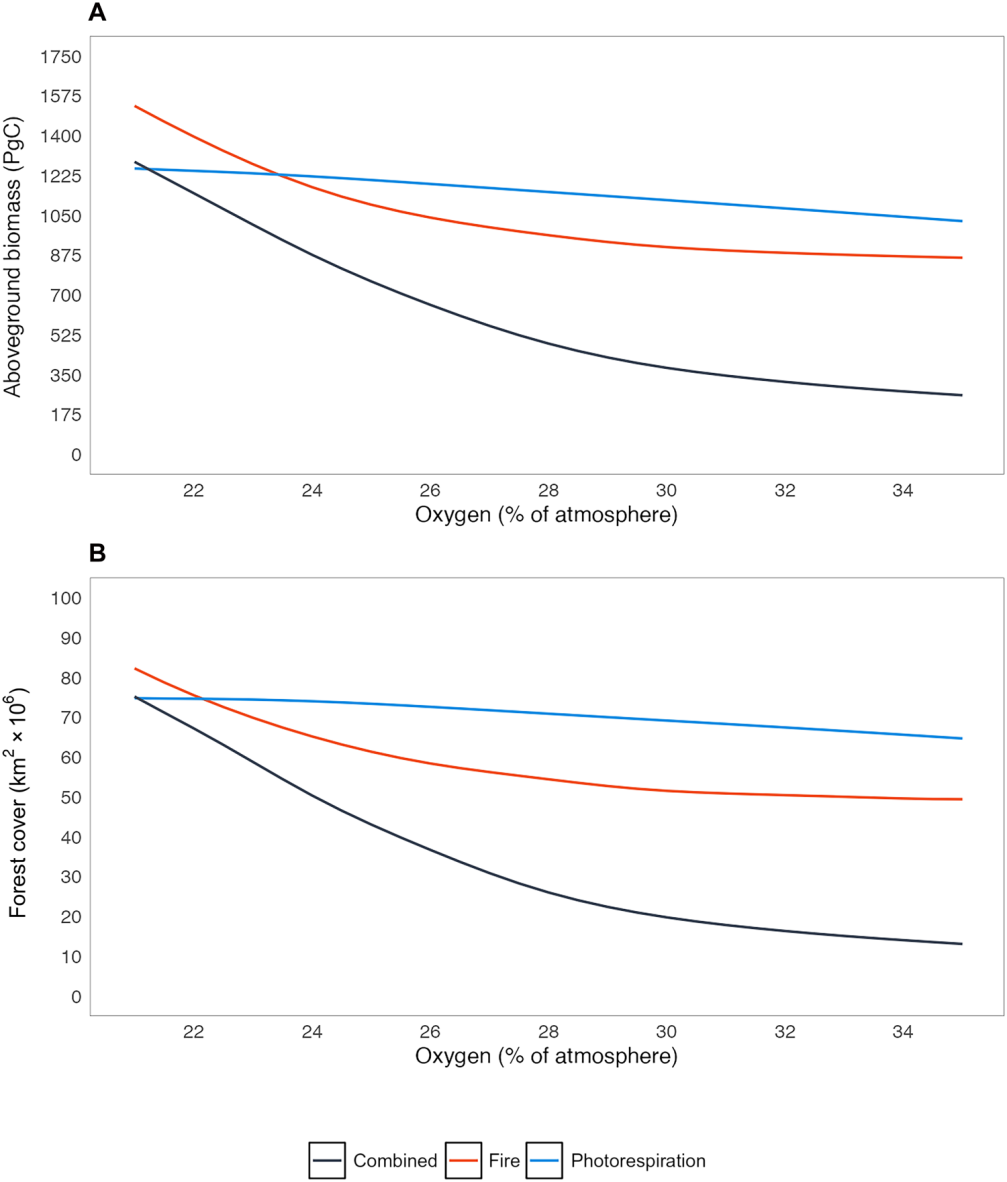
Simulated global total vegetation over atmospheric oxygen under elevated CO_2_, temperature, and precipitation. (**A**) Aboveground biomass (PgC) and (**B**) forest cover (tree cover >60%, km^2^). Lines indicate different oxygen simulations, which include oxygen-fire effects only (red), oxygen-photosynthesis effects only (blue), and both oxygen-fire and oxygen-photosynthesis effects (black). Totals are calculated from 10-year annual averages from LPJ-LMfire output, summed over grid cells to give a single global value.

**Fig. 4.**
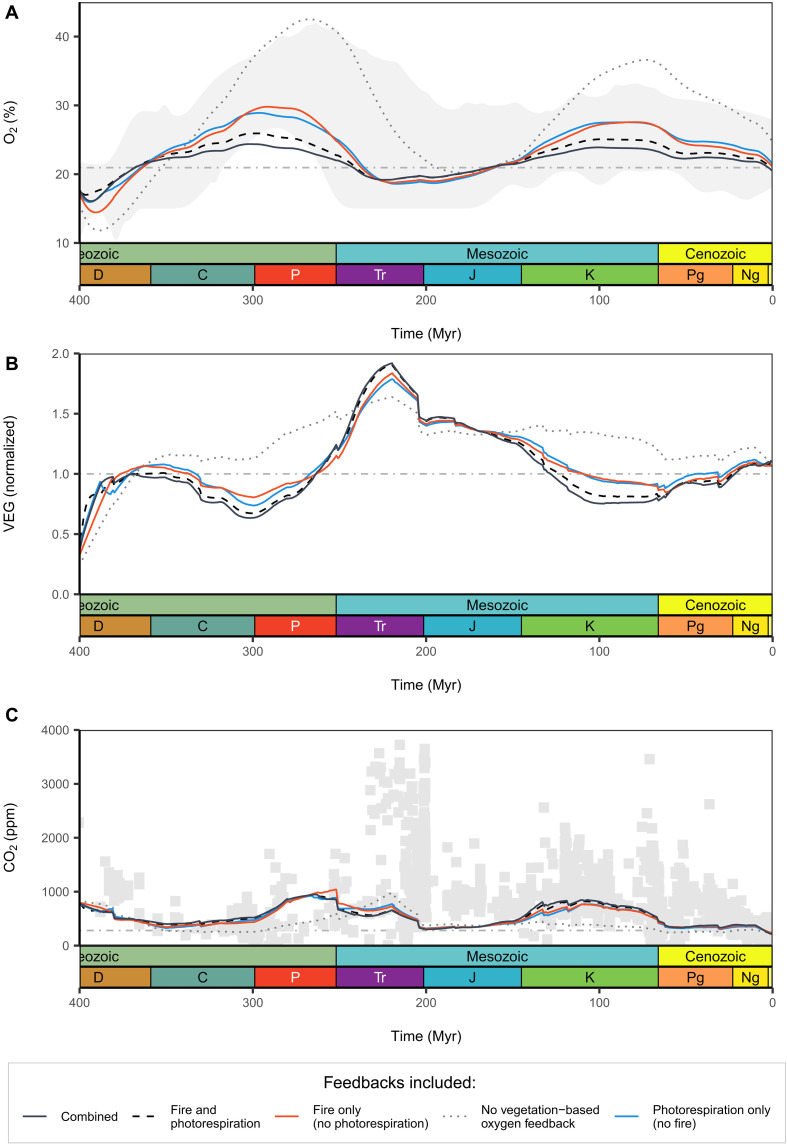
Vegetation-based negative feedbacks on atmospheric O_2_ in COPSE simulations. (**A**) Atmospheric O_2_, (**B**) terrestrial biomass, and (**C**) atmospheric CO_2_ under different feedback scenarios: no vegetation feedbacks (gray dotted), fire only (red), photorespiration only (blue), additive fire + photorespiration (black dashed), and coupled fire-photorespiration feedbacks (black solid). Horizontal gray lines mark present-day O_2_ (A), normalized biomass (B), and preindustrial CO_2_ (C). Shaded area in (A) shows the O_2_ consensus range from (*2*) and (C) CO_2_ proxy data (see fig. S4). Feedback terms are informed by LPJ-LMfire outputs (see Fig. 7 and Materials and Methods). ppm, parts per million.

Simulations conducted under alternative climate states, designed to reflect plausible Phanerozoic conditions, broadly support the patterns observed in the present-day climate runs (Figs. 3 and 4, figs. S6 to S10, and table S1). In scenarios with elevated CO_2_ alone [1000 parts per million (ppm)], photorespiration effects are globally weakened under high atmospheric O_2_ (figs. S6 and S9A, blue lines). Despite the overall reduction in vegetation suppression, the combined effects of fire and photorespiration still produce a greater-than-additive response, removing more than 60% of global biomass and confining forest cover to high latitudes [fig. S6C(c)].

To isolate the role of temperature, a separate configuration was run with elevated temperatures alone (without increased CO_2_, but including a lower meridional gradient). Under this scenario, both fire and photorespiration effects intensify with rising O_2_ concentrations (figs. S7, S9B, and S10B). Higher temperatures increase fire activity by drying moisture-limited regions and simultaneously enhance photorespiration rates—together amplifying vegetation loss across latitudes.

**Fig. 5.**
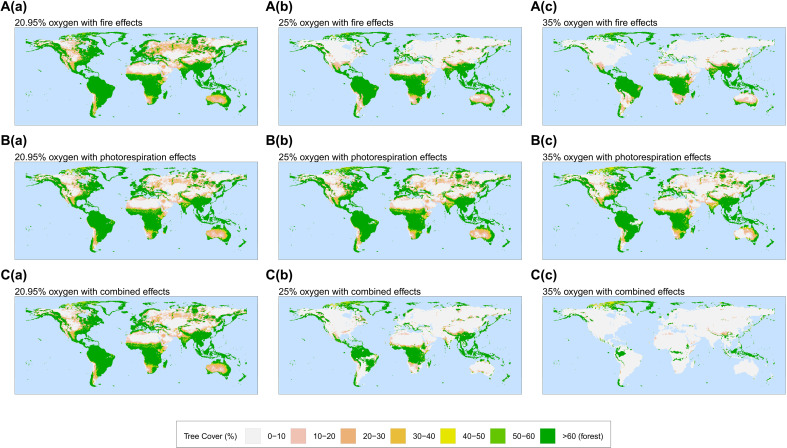
Plots of global tree cover (%) under elevated CO_2_, temperature, and precipitation. Subplots show (**A**) oxygen-fire effects only, (**B**) oxygen-photorespiration effects only, and (**C**) both oxygen-fire and oxygen-photorespiration effects, and output is plotted for (a) 20.95 vol % O_2_ (PAL), (b) 25 vol % O_2_ and (c) 35 vol % O_2_. Plots are taken as 10-year annual averages with forest cover defined to be tree cover greater than 60%. Here, elevated temperatures also include a lower meridional temperature gradient (for more details, see Materials and Methods).

The two climate scenarios combining elevated CO_2_ and temperature (one with increased precipitation and one without) yield similar outcomes (Fig. 5, fig. S8, and table S1). Elevated precipitation increases global biomass slightly by ~5% at PALs of O_2_, which leads to a very minor dampening of effects under high levels of atmopsheric oxygen (table S1). Figures 3 and 5 present simulation results under the elevated CO_2_, temperature, and precipitation climate configuration, for which under high O_2_ concentrations, vegetation losses are again substantial. Under 35 vol % O_2_, fire alone removes 44% of global biomass (Fig. 5A), primarily at mid-latitudes. Photorespiration has a somewhat weaker effect in this configuration, reducing global biomass by ~20% compared to ~60% under the default climate (blue lines in Figs. 3 and 5B), due to the dampening of photorespiration impacts in low-latitude regions caused by high CO_2_.Yet, when fire and photorespiration are combined, their interaction results in a >80% reduction in global biomass and forest cover [Fig. 5C(c) and table S1], suggesting that the suppression of vegetation by photorespiration at the lower latitudes is enough to remove vegetation by fire where fire cannot alone. These findings reinforce the conclusion that the joint fire-photorespiration feedback has a significantly stronger regulatory effect on vegetation than either mechanism alone and thus a greater potential to constrain atmospheric oxygen levels through geological time.

While this study primarily focuses on the effects of fire and photorespiration on atmospheric oxygen regulation and begins to look at different climate states, it is important to acknowledge that other factors, such as the different continental configuration and plant functional types that existed in Earth’s past, would also play notable roles in determining net terrestrial productivity and oxygen dynamics. The LPJ-LMfire model used in this study assumes a present-day continental configuration, with fixed latitudinal distributions and climatic belts. However, the position and configuration of continents have varied considerably over geological time, which would have affected the area available for different types of vegetation and the spatial distribution of fire and photorespiration feedbacks. For example, shifts in landmasses and the extent of tropical regions would likely influence the prevalence of wildfire feedbacks in certain areas and potentially modulate the temperature sensitivity of photorespiration. In addition, simulations presented here include modern-day plant functional types (PFTs), which introduce a limitation when analyzing impacts over certain periods over the Phanerozoic, where different vegetation types existed and in some cases those of today had not yet evolved (e.g., C4 grasses did not evolve until the Paleogene) These considerations highlight the importance of future work that could incorporate different continental configurations, climate states, and relevant plant PFTs to better understand their potential influence on oxygen regulation and terrestrial primary productivity throughout Earth’s history.

### Long-term regulation of atmospheric oxygen

**Fig. 6.**
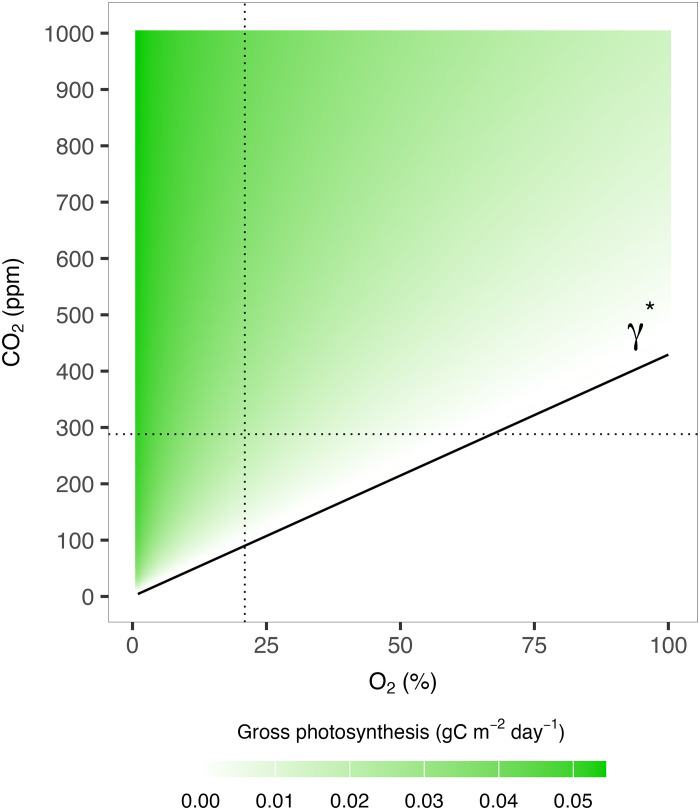
Compensation point (γ*) and gross photosynthesis from the LPJ-LMfire photosynthesis module over a range of CO_2_ and O_2_. Photosynthesis plotted as output from the LPJ-LMfire module for a set temperature of 20°C and dark respiration assumed to be 10% of total oxygen evolution (see Materials and Methods). The single compensation point (γ*) defined by André (*18*) used in this study is shown as a black solid line. Dotted lines show the preindustrial O_2_ and CO_2_ concentrations or 20.95% and 288 ppm, respectively.

**Fig. 7.**
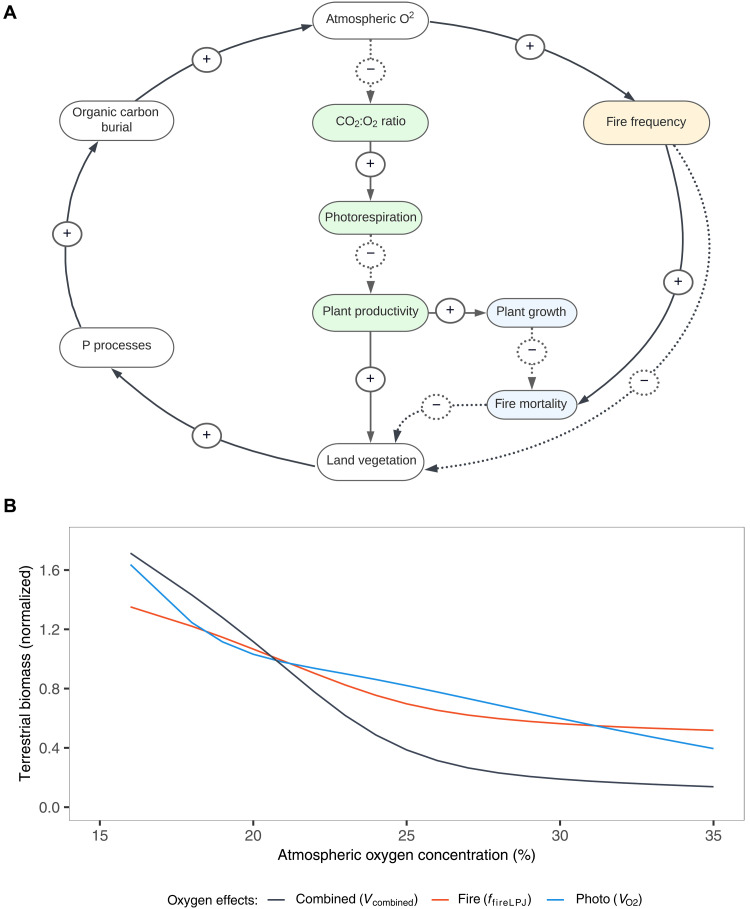
Feedback mechanisms and simulation results for atmospheric oxygen. (**A**) Diagram of proposed combined fire-photorespiration feedback on atmospheric oxygen. Positive feedbacks (solid arrows, “+”) indicate direct relationships, while negative feedbacks (dashed arrows, “−”) represent inverse relationships. Green boxes denote photorespiratory feedback, the orange box represents fire feedback, and blue boxes illustrate combined interaction effects. Here, the box labeled “P processes” refers to phosphorus redistribution from land to ocean and phosphorus weathering by faster generating vegetation [more details on specific feedbacks are given in (*1*)]. (**B**) Normalized terrestrial biomass as a function of atmospheric oxygen, derived from LPJ-LMfire simulations averaged globally over each oxygen level, used to update scaling factors in COPSE. Lines represent simulations with only oxygen-fire effects (red), only oxygen-photorespiration effects (blue), and combined effects (black).

“Forwards” or “predictive” biogeochemical models are commonly used to try to reconstruct the controls on atmospheric O_2_ over geological time and to predict the overall atmospheric O_2_ level through the Phanerozoic (*31*). The COPSE (Carbon-Oxygen-Phosphorus-Sulfur-Evolution) model (*31*) specifically includes the direct effects of reduced terrestrial biomass on carbon burial and the counter-effect of redistribution of phosphate to the oceans (*5*), both of which are dominant processes in atmospheric O_2_ feedbacks. The inclusion of negative vegetation-based feedbacks (predominantly through fire) exerts major control on the O_2_ predictions from these models (*1*, *9*, *31*, *35*), where exclusion of these feedbacks results in extremely high levels of O_2_ (e.g., gray-dotted line, Fig. 4) that tend to be outside of the boundaries set by available proxies (*1*). Through implementing the results for oxygen effects on vegetation from LPJ-LMfire into the COPSE biogeochemical model (see Materials and Methods), we found that including fire and photorespiration feedback effects singularly resulted in late Cretaceous oxygen peaks of 27.6 and 27.5 vol % O_2_, respectively (Fig. 6, counter effect). This is significantly lower than model runs excluding any vegetation-based negative feedbacks, which reached a maximum of ~37 vol % O_2_, rising 75% above PAL. The inclusion of the combined fire-photo respiration feedback proposed above (Fig. 7A) resulted in the tightest O_2_ regulation over Phanerozoic time, rising only 14% above PAL in the late cretaceous peak reaching just 23.8 vol % O_2_ (Fig. 4, black solid line). While including both updated fire and photorespiration feedbacks separately in the same simulation in COPSE (i.e., using the fire and photorespiration columns from table S5) resulted in weaker regulation of atmospheric oxygen through the Phanerozoic (Fig. 4, black dashed line). This indicates that combined fire-photorespiration effects that account for interactions between fire and photorespiration can provide considerably stronger negative feedback on atmospheric oxygen and therefore must be considered in discussions of major controls on O_2_ over geological time. It is only by using a dynamic vegetation model that these latitudinally distinctive and additive effects can be simulated, showing how the combination of effects can outweigh the sum of their individual strength.

The resulting prediction of Phanerozoic O_2_ using the updated combined fire and photorespiration feedback aligns broadly with previous studies and proxy data, showing two pronounced peaks: one in the late Carboniferous/early Permian and another in the late Cretaceous (*2*, *36*). The model’s tight regulation of O_2_ levels near PALs supports early theoretical calculations of an upper limit for atmospheric oxygen. Early work, including that of Lovelock (*37*) suggested that O_2_ concentrations exceeding ~25% by volume would lead to widespread wildfires, threatening forest regeneration (*1*, *7*). The near-continuous presence of forests in the fossil record supports this conclusion (*38*, *39*), indicating that O_2_ levels likely did not rise above this threshold. Subsequent studies, based on proxies and models, have proposed higher upper limits for atmospheric oxygen, arguing that increased fuel moisture content under elevated O_2_ could mitigate the impacts of widespread wildfires (*9*, *11*, *14*, *38*, *40*). However, these arguments have generally overlooked and underestimated the effects of photorespiration and fire-photorespiration-vegetation interactions, which may impose additional constraints on atmospheric oxygen levels. It is also important to highlight that other negative feedbacks not included in the current version of COPSE, such as a direct oxygen dependence of marine organic carbon preservation and burial, may act to further constrain atmospheric O_2_ variability, likely resulting in even tighter regulation and a lower maximum O_2_ peak through time.

The update to terrestrial feedbacks in the COPSE model has had minimal impact on reconstructions of metrics such as δ^13^C, which maintains a good fit with the geological record and proxy data originally used for COPSE model validation (see fig. S4). While the simulated evolution of atmospheric O_2_ aligns with the general patterns derived from the charcoal record [e.g., figure 5A in (*2*)], the absolute O_2_ values are at times lower than those inferred from some proxies and recent modeling studies, for example, against the consensus O_2_ curve range suggested by Mills *et al.* (*2*) range and shown in Fig. 4A. This discrepancy can be attributed to the calibration of inertinite data, where maximum inertinite levels are traditionally scaled to assumed upper limits of 30 to 35 vol % O_2_ (*9*, *38*, *41*). If a lower upper limit of atmospheric oxygen, as argued in this study, were applied, then the proxy-derived O_2_ range would also shift downward, improving agreement with the presented simulation. Moreover, many biogeochemical models have been validated using charcoal-based proxies with these previously assumed limits, further compounding this circularity (*2*, *31*, *32*, *42*, *43*). This highlights a critical limitation: Atmospheric O_2_ predictions from some proxies are influenced by bounds derived from earlier model assumptions, while those same models are validated against these proxies. Consequently, comparisons between proxies and models must be interpreted with caution.

The results presented in the figure align broadly with previous modeling studies. However, the work presented here offers an important contribution by addressing key knowledge gaps in the mechanisms underlying atmospheric oxygen regulation. While prior studies have explored Phanerozoic oxygen dynamics and the general O_2_ trajectory (*2*, *11*), the testing of specific feedbacks influencing oxygen levels over time remains underexplored. Recent advances, such as the use of DGVMs and Earth system models, have started to investigate these mechanisms, for instance, to examine O_2_ impacts on climate (*36*). However, how Earth system and climate feedbacks collectively influence oxygen concentrations remains poorly understood. This study investigates the spatial interactions of two critical processes—fire and photorespiration—in regulating atmospheric oxygen concentrations through terrestrial feedbacks. By leveraging DGVMs, the study integrates spatial variability, highlighting how these processes interact under varying climatic and environmental conditions. Specifically, it shows that where fire feedbacks are dampened by high moisture levels, the temperature sensitivity of photorespiration becomes a dominant regulatory mechanism, particularly in tropical ecosystems. This spatially explicit approach, not previously applied in this context, advances our understanding of the coregulation mechanisms stabilizing atmospheric oxygen and provides a refined framework for interpreting proxy records and evaluating assumptions in earlier modeling studies.

While this study has begun to explore spatial interactions between oxygen, CO_2_, and temperature on photorespiration and fire feedbacks, future work implementing a comprehensive matrix of climate and atmospheric gas scenarios in the DGVM could provide mechanistic insights to better inform and refine the vegetation feedback parameterizations within the COPSE model. This would enable a more integrated treatment of coupled CO_2_─O_2_ dynamics and their influence on compensation points, advancing our understanding of biosphere-atmosphere interactions and their role in long-term atmospheric oxygen regulation.

We have demonstrated that the combination of oxygen-fire and oxygen-photorespiration feedback effects likely results in extreme limitation of global terrestrial biomass when atmospheric O_2_ levels rise far above PAL. Thus, we conclude that atmospheric oxygen concentration on Earth is very tightly regulated, more so than has traditionally been considered, and perhaps to the point where it has remained in the range of 21 to 24% over the past 150 Myr. This stability has important consequences for the evolution of animal ecosystems: the high O_2_ levels that supported mammals may have been in place for hundreds of millions of years before they evolved (*2*), and stable and high O_2_ levels may have contributed to the lower extinction rates of the past 400 Myr compared to the high turnover in the Early Paleozoic (*36*, *44*).

## MATERIALS AND METHODS

### LPJ-LMfire model summary

We used the LPJ-LMfire DGVM, a version of the LPJ model (*45*) with enhanced details of fire dynamics, built for simulating interactions between climate, vegetation, and fire regimes during prehistoric and preindustrial times (*22*). The model incorporates nine PFTs to represent different vegetation groups, simulating their growth, reproduction, and mortality. The model uses input data (see table S1) including climate (such as temperature, CO_2_ concentrations, and precipitation), soil, and topography data together with vegetation characteristics. These inputs are used to drive processes of photosynthesis, respiration, and biomass allocation, while soil moisture and nutrient availability influence plant growth and competition. Fire dynamics are modeled by simulating fire ignition, fire spread, intensity, and severity, which are affected by fuel load, moisture content, and weather conditions. Biogeochemical cycles are also included where carbon and nitrogen fluxes are tracked within ecosystems. Simulations run on a 0.5° spatial resolution and designed to operate on a daily time step. Outputs include vegetation distribution, fire regime metrics, carbon and nutrient fluxes, and water dynamics, providing insights into the ecological impacts of fire.

We updated LPJ-LMfire to include a parameter for O_2_ concentration, which was altered to influence fire and photorespiration simulated in the model. The model parameter for O_2_ concentration was set as an external input and therefore remained fixed at a predetermined value throughout all simulations. This setup ensures consistency in modeling the interactions between fire dynamics, photorespiration, and forest coverage and allows for a controlled investigation of how fixed levels of O_2_ affect ecosystem dynamics, providing clear insights into the influence of these gases under the given simulation scenarios. Hence, through introducing an atmospheric oxygen parameter and including relationships between oxygen and both fire and photosynthesis, we were able to analyze natural oxygen-vegetation effects under a range of oxygen concentrations through a series of simulations.

Changes to fire and photorespiration processes within the model are described in the subsections below, followed by a description of the oxygen simulation experiments conducted using LPJ-LMfire. For a full model description of LPJ-LMfire, see (*22*).

### Fire

Within LPJ-LMfire, fire is based on the SPITFIRE (SPread and InTensity of FIRE) process-based model (*46*) but includes numerous improvements to the representation of fire including multi-day burning and coalescence of fires and explicit calculation of natural ignitions (*22*). As a necessary component of fire, the abundance of oxygen in the atmosphere influences fire ignition and behavior. Numerous studies have found that the probability of ignition and rate of fire spread increases sharply with rising oxygen concentrations (*7*, *11*–*13*), while growing evidence suggests that the energy released from a fire in the form of heat (heat of combustion) is also dependent on oxygen (*47*). We therefore alter the fire module in LPJ-LMfire following (*14*), such that key components of fire behavior (probability of ignition, moisture of extinction, and heat of combustion) are simulated to vary under different atmospheric oxygen concentrations.

Within the LMfire fire module, ignition efficiency (ieff) is defined as the product of the fire danger index calculated based on relative fuel moisture and fuel type, the average ignition efficiency of vegetation within the gridcell (${\text{ieff}}_{\text{avg}}$), and previous burned area within the gridcell (${\text{ieff}}_{\text{bf}}$), such that the likelihood of an ignition occurring decreases based on an increase in area already burned to date

$$\text{ieff}=\text{FDI}\cdot {\text{ieff}}_{\text{avg}}\cdot {\text{ieff}}_{\text{bf}}$$ (1)

The average ignition efficiency is calculated as a weighted average based on foliar projected cover ($\text{fp}c_{\text{grid}}$) and individual ignition efficiencies (${\text{ieff}}_{\text{pft}}$) of each pft, where ignition efficiencies are based on prescribed constants

$${\text{ieff}}_{\text{avg}}=\frac{\sum_{\text{pft}}^{\text{npft}}\left(\text{fp}c_{\text{grid}}\cdot {\text{ieff}}_{\text{pft}}\right)}{\sum_{\text{pft}}^{\text{npft}}\text{fp}c_{\text{grid}}}$$ (2)

Here, oxygen-dependent probability of ignition is added through scaling individual ignition efficiency constants based on atmospheric oxygen concentration prescribed to the model using probability of ignition as a function of atmospheric oxygen concentration (O_2_) and fuel moisture content (M), taken from (*7*)

$$\begin{array}{c} \text{PI}\left(O_{2},M\right)=\left[308.02-27.406\left(O_{2}\right)+0.634{\left(O_{2}\right)}^{2}-0.0044{\left(O_{2}\right)}^{3}\right]\text{ln}\left(M\right)\\ -633.54+42.327\left(O_{2}\right)-0.2194{\left(O_{2}\right)}^{2}-0.0075{\left(O_{2}\right)}^{3} \end{array}$$ (3)

Because fuel moisture within the model is divided into woody fuel moisture ($\omega_{o}$) and 1-hour fuel and live grass moisture ($\omega_{\text{nl}}$), ignition efficiency based on oxygen level is calculated as

$${\text{ieff}}_{ox_{g}}=\frac{\text{PI}\left(O_{2},\omega_{\text{nl}}\right)}{\text{PI}\left(20.95,\omega_{\text{nl}}\right)}$$ (4)

$${\text{ieff}}_{ox_{w}}=\frac{\text{PI}\left(O_{2},\omega_{o}\right)}{\text{PI}\left(20.95,\omega_{o}\right)}$$ (5)

where ${\text{ieff}}_{ox_{g}}$ and ${\text{ieff}}_{ox_{w}}$ are the scaled ignition efficiencies due to oxygen for grasses and woody fuels, respectively, normalized around PALs (20.95 vol % O_2_). Therefore, if oxygen-fire effects are switched on in the model, then Eq. 2 becomes

$${\text{ieff}}_{\text{avg}}=\frac{\sum_{\text{pft}}^{\text{npft}}\left(\text{fp}c_{\text{grid}}\cdot {\text{ieff}}_{\text{pf}t_{\text{ox}}}\right)}{\sum_{\text{pft}}^{\text{npft}}\text{fp}c_{\text{grid}}}$$ (6)

where

$${\text{ieff}}_{\text{pf}t_{\text{ox}}}=\left\{\begin{array}{c} {\text{ieff}}_{\text{pft}}\cdot {\text{ieff}}_{ox_{g}},\text{pft}=\text{grass}\\ {\text{ieff}}_{\text{pft}}\cdot {\text{ieff}}_{ox_{w}},\text{pft}=\text{tree} \end{array}\right.$$ (7)

The moisture of extinction describes the limit of fuel moisture content that prevents fire spread and is therefore critical in determining fire within LPJ-LMfire. Within the model, constants for moisture of extinction are prescribed for each fuel class before a mass-weighted average is calculated for each gridcell ($M_{\text{avg}}$). Combustion experiments show that the moisture of extinction increases with rises in atmospheric oxygen (*7*). We therefore introduce an equation for moisture of extinction dependent on atmospheric oxygen ($M_{e}$) as described in (*7*)

$$M_{e}=8O_{x}-128$$ (8)

which is then normalized around PALs to give a scale factor of moisture of extinction based on levels of atmospheric oxygen ($M_{e\_\text{ox}}$)

$$M_{e\_\text{ox}}=\frac{M_{e}\left(O_{2}\right)}{M_{e}\left(20.95\right)}$$ (9)

Therefore, the overall moisture of extinction for each gridcell (me) becomes

$$\text{me}=M_{\text{avg}}\cdot M_{e\_\text{ox}}$$ (10)

Last, growing literature suggests that the heat of combustion (amount of energy released in a fire in the form of heat) is dependent on both atmospheric oxygen concentration and vegetation type (*47*–*49*). Previously, LPJ-LMfire included a set value of 18,000 kJ g^−1^ for the heat of combustion, irrespective of PFT. We therefore include the improved version of heat of combustion as outlined in (*14*), although the addition of an equation for heat of combustion ($h_{\text{pft}}$) is dependent on PFT type and atmospheric concentration

$$h_{\text{pft}}=\frac{\alpha_{\text{pft}}}{O_{x}}+\beta_{\text{pft}}$$ (11)

where $\alpha_{\text{pft}}$ and $\beta_{\text{pft}}$ are PFT coefficients outlined in table S3. The overall heat of combustion in the grid cell is then calculated as a single foliage projected cover–weighted average ($h_{\text{avg}}$)

$$h_{\text{avg}}=\frac{\sum_{\text{pft}}^{\text{npft}}\left(\text{fp}c_{\text{grid}}\cdot h_{\text{pft}}\right)}{\sum_{\text{pft}}^{\text{npft}}\text{fp}c_{\text{grid}}}$$ (12)

### Photorespiration

The photosynthesis scheme within LPJ-LMfire is adapted from the Farquhar and Caemmerer model (*26*), as simplified by Collatz *et al.* (*50*, *51*) and Haxeltine and Prentice (*52*). The daily photosynthesis is calculated as a function of absorbed photosynthetically active radiation (APAR), atmospheric CO_2_ concentration, temperature, canopy conductance, and day length and is determined by two limiting rates: the response of photosynthesis to APAR and the limitation of Rubisco activity.

Within the model, the atmospheric oxygen concentration affects the Rubisco-limited rate of photosynthesis through inclusion of a CO_2_ compensation point, defined to be the CO_2_ concentration at which the net CO_2_ fixation is zero for constant O_2_ concentration (set at PAL) and temperature, taken from (*53*). While this is sufficient for model use with oxygen assumed to be constant at PAL, research since original definitions of compensation points has found that, due to the dual activity of Rubisco, an O_2_ compensation point exists that defines the equilibrium point between photosynthesis and the opposite respiratory processes for a given temperature and CO_2_ level (*16*–*18*). Therefore, to simulate the effect of varying oxygen on photorespiration, an update of the compensation point in the model was required.

Over recent decades, various studies have attempted to formulate the impact of varying O_2_ on rates of photorespiration and photosynthesis including Tolbert *et al.* (*17*) definitions of separate compensation points and Nisbet and Nisbet (*16*) who suggested the concept of a permitted zone. Others have conducted experimental and modeling studies that support the existence of an O_2_ compensation point (*15*, *20*, *54*). A notable study was conducted by André (*18*), who—through a critical analysis of both their own work and others—found that a reciprocal relationship exists between CO_2_ and O_2_ compensation points [see figure 5 in (*18*)], such that a single function defining both exists.

We therefore update LPJ-LMfire to include an approximated single compensation point that varies over O_2_ using equation 12B, section 4.5, from (*18*)

$$\gamma^{*}=\delta \cdot \frac{O_{2}}{\tau }$$ (13)

where O_2_ is the atmospheric oxygen concentration (%) and δ is a fraction dependent on rates of dark respiration assumed. Here, we assume moderate but not excessive dark respiration, taken to be 10% of total oxygen evolution, such that $\delta =\frac{2}{3}$ [R = 0.1E used in equation 12B in (*18*)]. τ is the CO_2_/O_2_ specificity factor and is defined by a q10 relationship for a given temperature, T, as

$$\tau =\tau_{25}\cdot q10_{\tau }^{\left(\frac{T-25}{10}\right)}$$ (14)

Where $\tau_{25}$ and $q10_{\tau }$ are coefficients with values 2600 and 0.57, respectively. The addition of this results in an intermediate photorespiration effect that can allow net photosynthesis to remain positive up to a very high oxygen concentration (see Fig. 6) and is applied to PFTs in each grid cell dependent on simulated temperature and prescribed CO_2_ and O_2_ concentrations. A full description of the photosynthesis module can be found in (*52*).

### Oxygen simulations

To analyze the effect of atmospheric oxygen on vegetation through fire and photosynthesis, a series of LPJ-LMfire simulations were run at different oxygen levels ranging from 20.95 to 35 vol % O_2_, in accordance with the range of validity of the experiments from which changes to the probability of ignition were based (*7*). The complete series of O_2_ simulations were undertaken for different configurations of the model, namely, fire updates only (where only fire is dependent on O_2_ concentration), photorespiration only (only photorespiration depends on O_2_ concentration), and both, where both fire and photorespiration are dependent on O_2_ concentrations. Simulations were driven using detrended and transient reanalysis data spanning a 40-year period (1971–2010; see table S2), where the dataset of climatic inputs is repeatedly cycled over the simulation period. For any given simulation, O_2_ concentration is set at a predetermined constant and therefore, as with CO_2_, is not dynamically recalculated in LPJ-LMfire but treated as external input. As oxygen has not varied considerably during the time of human existence, and since we were interested in natural relationships with oxygen, simulations were run with humans excluded so that no human influence on vegetation or fire was modeled and only natural, lightning-caused fires could start. All simulations conducted were initialized for 1500 years to ensure an equilibrium was reached, with results taken from the past 10 years of the simulation taken as output, plotted as 10-year annual averages.

A very high fraction of wildfires in the modern world is largely influenced by human activity (suppression, ignition, etc.); hence, evaluating the model’s ability to simulate natural fire and vegetation against observations is difficult at a global scale. In regions where natural fire is still dominant, such as boreal and subarctic regions, LPJ-LMfire has been extensively evaluated and found to be in much better agreement with observations than previous model versions (*22*). To ensure the model is still valid with the changes to fire and photorespiration as above, we compared model simulations at 20.95 vol % O_2_, with both the changes for photorespiration and fire included, against previous model simulations (without changes) and observations in Alaska. While simulations show a general slight overestimation in aboveground biomass compared to observational data for this region (see fig. S5), this overestimation of biomass is noted in previous, published versions of the model [see figure 1A in (*22*)], and thus changes implemented did not significantly affect results. Similarly, burned area simulated in Alaska was not affected by the model changes and was consistent with previous model versions and observations [see table 4 and figure 5 in (*22*)], where Intermontane Boreal ecoregions are seen to have elevated burned area, averaging more than 0.8% of each grid cell (fig. S5C).

Simulations were also conducted to evaluate the model’s sensitivity to various parameters used in this study. These parameters include moisture of extinction and heat of combustion, both related to fire behavior, together with specificity factor and dark respiration rates for photorespiration. To thoroughly assess sensitivity, an ensemble of simulations was performed, encompassing all possible combinations of minimum and maximum values for the four key parameters. The realistic range for these parameters was defined on the basis of existing literature and are presented in table S4 and described in Supplementary Methods. These sensitivity simulations followed the same protocol as the primary simulations described above, where decadal averages were calculated from the final 10 years of each 1500-year-long simulation.

### Climate simulations

Throughout the Phanerozoic, periods of elevated atmospheric oxygen have often coincided with high CO_2_ concentrations and warmer global temperatures (*2*, *6*, *25*, *31*), while some intervals are thought to have also experienced a wetter climate, such as the Late Cretaceous (*55*). To test the robustness of our findings under different climatic conditions, we repeated the oxygen-fire-photorespiration simulations described above using LPJ-LMfire across four alternative climate scenarios. These scenarios represent plausible Phanerozoic climate extremes associated with high atmospheric oxygen and include: (i) elevated CO_2_; (ii) elevated temperature; (iii) combined elevated CO_2_ and temperature; and (iv) elevated CO_2_, temperature, and precipitation.

Elevated CO_2_ levels were prescribed directly in the model at 1000 ppm. For high-temperature scenarios, input temperature fields were adjusted on the basis of the meridional scaling relationships described in (*56*), applying a latitudinally dependent gradient consistent with greenhouse climates. In the high-precipitation configuration, modeled precipitation was increased uniformly by 25% based on estimates from the literature (*55*). The full set of oxygen simulations was repeated for each of these four climate scenarios. Results from the combined elevated CO_2_-temperature-precipitation scenario are presented in the main text, with the outcomes of the other scenarios provided in the Supplementary Materials.

### COPSE model summary

To test the implications that the different oxygen-vegetation effects may have on long-term oxygen regulation, we run simulations using the COPSE model (*19*). COPSE is a simple nondimensional biogeochemical box model designed to simulate Earth system interactions over geological timescales. The model represents global biogeochemical cycles of carbon, oxygen, phosphorus, and sulfur. It considers how changes in temperature, nutrient availability, and biological productivity feedback onto biogeochemistry, predicting the coupled histories and controls on atmospheric O_2_, CO_2_, and ocean composition over Phanerozoic time (*31*, *43*).

Originally, COPSE was developed by Bergman *et al.* (*19*) and was based on the GEOCARB models (*57*, *58*) but differs in that it uses a forward’s model approach. Forward models aim to explicitly simulate biogeochemical fluxes such as organic carbon burial rather than driving this from isotope records, allowing for easy representation of feedback processes and leaving proxy data for comparison and revisions (*1*). The model uses a single box to represent the atmosphere and ocean with sedimentary inventories and key reservoirs, such as carbon and oxygen, represented as a series of boxes with initially prescribed sizes, which are then updated iteratively based on fluxes (representing processes) between them.

COPSE is driven by several “external forcings” including time-dependent solar insolation, volcanic and tectonic activity, and evolutionary switches [for a full list of forcings and prescribed reservoir sizes, see the Supplementary Materials in (*43*)] and is computed in MATLAB using a variable order ordinary differential equation solver. Since the original model, numerous versions of COPSE have been developed. Here, we use a recent release based on the model that is lightly updated from (*43*). A full model description together with source code and forcing data can be found at https://github.com/bjwmills/COPSE.git.

In contrast to LPJ-LMfire, the COPSE model internally calculates both atmospheric O_2_ and CO_2_ at each time step. Both atmospheric O_2_ and CO_2_ are represented in COPSE by a single global value. Atmospheric CO_2_ is taken to be a fraction of the total ocean-atmosphere reservoir and is influenced by external forcings (solar insolation, tectonic input, etc.), biological activities and evolutionary changes, and geochemical processes (for instance, burial and weathering rates) that alter the total carbon in the model. Similarly, atmospheric oxygen varies depending on the long-term O_2_ sources (organic carbon and pyrite sulfur burial) and sinks (uplift and weathering or subduction and degassing) (*43*). In earlier versions of COPSE, an explicit oxygen dependence was included in the marine organic carbon burial (mocb) flux, introducing a direct negative feedback on atmospheric O_2_ levels (*31*). This feedback was found to produce smaller O_2_ variations and slightly tighter regulation through time. While conceptually realistic, its inclusion shifted modeled O_2_ trends away from geochemical proxy records. Consequently, this oxygen-dependent mocb feedback is not included in the present version of COPSE used here. An indirect O_2_-marine feedback remains in our simulations: Phosphorus burial fluxes are retained with an inverse dependence on ocean anoxia, such that more reducing conditions enhance P recycling (*31*, *43*). While COPSE incorporates a number of other feedbacks [see (*31*, *43*) for more details], we focus here on the terrestrial vegetation-fire-oxygen feedback that has been found to have the largest impact on atmospheric oxygen control in COPSE simulations (*31*). In this interaction, changes in atmospheric O_2_ affect fire activity and vegetation cover, which in turn influence the burial of organic carbon and thus atmospheric O_2_ itself.

Because of the coupling of components in COPSE, this means that not only do the changes in CO_2_ and O_2_ affect the value calculated for terrestrial vegetation in the model, but any changes to vegetation through other processes (for instance, fire) will feedback onto atmospheric O_2_ and CO_2_.

Within the model, terrestrial biomass (V) is represented by a single global value, which is calculated as the product of terrestrial net primary productivity ($V_{\text{npp}}$) and a “fire regulation” parameter ($f_{\text{fire}}$), a normalized representation of the amount of fire suppression on vegetation for a given oxygen concentration

$$V=V_{\text{npp}}\cdot f_{\text{fire}}$$ (15)

where

$$f_{\text{fire}}=\frac{k_{\text{fire}}}{k_{\text{fire}}-1+\text{ignit}\left({\text{mO}}_{2}\right)}$$ (16)

Here, $k_{\text{fire}}$ is a constant ($k_{\text{fire}}=3$) following (*12*), such that fire suppression on vegetation equates to 50% under PALs relative to vegetation with no fires, assumed to be at ${\text{mO}}_{2}$ < 0.19 (volumetric mixing ratio). Function ignit(${\text{mO}}_{2}$) then scales the fire regulation parameter dependent, increasing linearly with atmospheric oxygen

$$\text{ignit}\left({\text{mO}}_{2}\right)=\text{min}\left[\text{max}\left(48\cdot {\text{mO}}_{2}-9.08,0\right),5\right]$$ (17)

While terrestrial net primary productivity is calculated based on an “OCT” (where OCT refers to the specific productivity formulation to calculate plant productivity dependent on O_2_, CO_2_, and temperature) formulation (*19*), such that it is dependent on temperature and both CO_2_ and O_2_ concentrations, which each have multiplicative factors within the model ($V_{T}$, $V_{\text{CO}2}$, and $V_{O2}$, respectively) and also being forced by a plant evolution and land colonization forcing factor, E

$$V_{\text{npp}}=2\cdot E\cdot V_{O2}\cdot V_{\text{CO}2}\cdot V_{T}$$ (18)

While $V_{\text{CO}2}$ describes the growth rate dependence on CO_2_ fertilization and $V_{T}$ describes the parabolic relationship between temperature and productivity, $V_{O2}$ describes oxygen inhibition of primary productivity through respiration as a linear relationship dependent on O_2_

$$V_{O2}=1.5-\left(0.5\cdot O_{2}\right)$$ (19)

### Phanerozoic simulations

We conduct Phanerozoic simulations in COPSE to analyze the impacts of the different oxygen-vegetation effects on long-term atmospheric oxygen regulation. The series of oxygen simulations conducted in LPJ-LMfire are analyzed to give values of annual global total biomass simulated under each oxygen concentration. Normalizing the global biomass output from LPJ-LMfire indicates how the different oxygen-vegetation processes (fire, photorespiration, and both) differ in their ability to scale suppress global vegetation under different O_2_ concentrations.

Within this study, we therefore replace normalized variables for global fire suppression and photorespiration within COPSE ($f_{\text{fire}}$ and $V_{O2}$) with normalized forcing factors $f_{\text{fireLPJ}}$ and $V_{O2\text{LPJ}}$, respectively, computed using LPJ-LMfire simulations above, such that terrestrial biomass within the model becomes

$$V=\left(k_{\text{npp}}\cdot E\cdot V_{O2\text{LPJ}}\cdot V_{\text{CO}2}\cdot V_{T}\right)\cdot f_{\text{fireLPJ}}$$ (20)

where forcings are normalized and interpolated from global total values of terrestrial aboveground biomass output from LPJ-LMfire for a given O_2_ concentration (see table S5).

Simulations were then run to include different combinations of vegetation-based feedbacks to analyze regulation on atmospheric oxygen (see overview in Table 1) including: no vegetation–based oxygen feedbacks (where both $V_{O2\text{LPJ}}$ and $f_{\text{fireLPJ}}$ were set to one, effectively switching them off), oxygen-fire feedback only ($V_{O2\text{LPJ}}=1$), oxygen-photorespiration feedback only ($f_{\text{fireLPJ}}=1$), both oxygen-fire and oxygen-photorespiration feedback included separately in the same run, and lastly a new combined fire-photorespiration feedback that accounts for interaction effects between fire and photorespiration (see Fig. 7A). In the combined-feedback simulation, $V_{O2\text{LPJ}}$ and $f_{\text{fireLPJ}}$ were set to one, and a new combined-effects forcing was added on the basis of LPJ-LMfire output, $V_{\text{comb}}$, such that Eq. 6 becomes

$$V=k_{\text{npp}}\cdot E\cdot V_{\text{comb}}\cdot V_{\text{CO}2}\cdot V_{T}$$ (21)

**Table 1.** Outline for configurations used in COPSE model feedback simulations over the Phanerozoic.

Simulation (feedback included)	$V_{O2}$	$f_{\text{fire}}$
No vegetation–based oxygen feedback	1	1
Photorespiration only (no fire)	$V_{O2\text{LPJ}}$	1
Fire only (no photorespiration)	1	$f_{\text{fireLPJ}}$
Fire and photorespiration	$V_{O2\text{LPJ}}$	$f_{\text{fireLPJ}}$
Combined	1	$V_{\text{combined}}$

## Supplementary Materials

### This PDF file includes:

Supplementary Methods Supplementary Text Figs. S1 to S10 Tables S1 to S5 References
