# Supplementary material for: Combined effects of photorespiration and fire strongly regulate atmospheric oxygen levels
Source: Sci Adv. 2026 Jan 7;12(2):eady0542. doi: 10.1126/sciadv.ady0542 (PMC12778039; doi:10.1126/sciadv.ady0542)
Supplement: Supplementary file 1 — Supplementary Methods Supplementary Text Figs. S1 to S10 Tables S1 to S5 References [file sciadv.ady0542_sm.pdf]

Science Advances
AAAS

Supplementary Materials for

Combined effects of photorespiration and fire strongly regulate atmospheric oxygen levels

Rayanne Vitali *et al.*

Corresponding author: Rayanne Vitali, rvitali@envs.au.dk

Sci. Adv. **12**, eady0542 (2026)
DOI: 10.1126/sciadv.ady0542

This PDF file includes:

Supplementary Methods
Supplementary Text
Figs. S1 to S10
Tables S1 to S5
References

Supplementary methods

Sensitivity analysis

Here, details regarding parameter sensitivity analysis described in the materials and methods are outlined. Simulations were conducted where all combinations of minimum and maximum values for four parameters (MoE, HoC, specificity coefficient and rate of dark respiration) were simulated. Minimum and maximum values were determined using literature and are described below and shown in Table S4.

Moisture of Extinction (MoE)

MoE is defined in the model as a linear function of atmospheric oxygen concentration (equation 8). Watson and Lovelock (*7*) propose fire cannot ignite and spread at 16% vol. O_2 regardless of moisture (i.e. MoE = 0), however other studies have argued that the lower limit of fire falls between 12-18% vol. O_2, therefore setting a lower range where MoE is zero (*1, 11-12*). For higher concentrations of O_2, Watson and Lovelock (*7*) already assume an extreme case where combustion is possible even under high levels of fuel moisture, whilst others have argued for lower MoE. For example, Wildman et al. (*11*) found that fuel moisture contents higher than 60% were unable to ignite and spread under 35% vol. O_2. A range of variability could then be set through assuming linear bounds using these ranges (illustrated in Fig. S2).

Heat of Combustion (HoC)

HoC is given for each PFT in the model (see Materials and Methods & Table S3 and Vitali et al. (*14*). Minimum and maximum linear limits are set using the compiled data across all PFTs used in Vitali et al. (*14*), shown in Fig. S2.

Specificity Coefficient (τ_{25})

A coefficient of 0.132 is used in the calculation of specificity factor, τ, taken from the average specificity factor found across plants in André (*18*). Here, the minimum and maximum values of specificity factor found across the plants in the study are used to set the range to test over (see Table.1 in André et al. (*18*)).

Rates of dark respiration (δ)

The factor of δ=2/3 used in the model corresponds to dark respiration equating to 10% of total oxygen evolution (see Methods). Yet literature suggests that rates of dark respiration can vary depending on various factors such as plant species and environmental conditions and can therefore range widely between 5-30% of photosynthetic rates (*33-4*). Therefore, using the same approach outlined in the Methods, new factors are derived using this range.

Supplementary text

The Strength of the oxygen-photorespiration feedback

In the main text we explore the effects of varying atmospheric oxygen levels on global vegetation using LPJ-LMfire through fire and photorespiration effects, using results to test strengths of potential feedbacks in COPSE. We therefore run simulations in LPJ-LMfire with constant (preindustrial) temperatures and CO_2 levels to examine the effects of changing oxygen alone. Yet, oxygen inhibition of photosynthesis is also dependent on temperature and CO_2 values. We might therefore expect a greater variation in the strength of the oxygen-photorespiration negative feedback than we have shown here. Increasing CO_2 above preindustrial levels would increase photosynthesis and reduce photorespiration as CO_2:O_2 ratio would move further above the compensation point (γ^*, Fig.7), whilst decreasing CO_2 would enhance photorespiration and lower gross photosynthesis until the CO_2:O_2 ratio reaches the compensation point (γ^*), below which plants will be unable to complete their life cycles (*15–17*). However, temperatures also strongly influence compensation points and productivity, with increased temperatures driving higher rates of photorespiration (*16–18*). This is significant because over the large parts of the Phanerozoic global temperatures have been higher than the present day (*31*). Hence, whilst we show that photorespiration and fire-photorespiration effects can provide strong regulation on atmospheric oxygen through substantially affecting global vegetation using the COPSE model, the specific strength of the feedback on global vegetation at any given time over the Phanerozoic requires further exploration of CO_2 and temperature effects which is not explored here in LPJ-LMfire. However, COPSE accounts for CO_2 and temperature effects on terrestrial biomass through variables that effectively scale the impact that photorespiration has on net primary productivity (see methods). Therefore, runs over geological timescales testing feedbacks in COPSE account for all factors affecting the compensation point and hence impacts on primary productivity through inhibition.

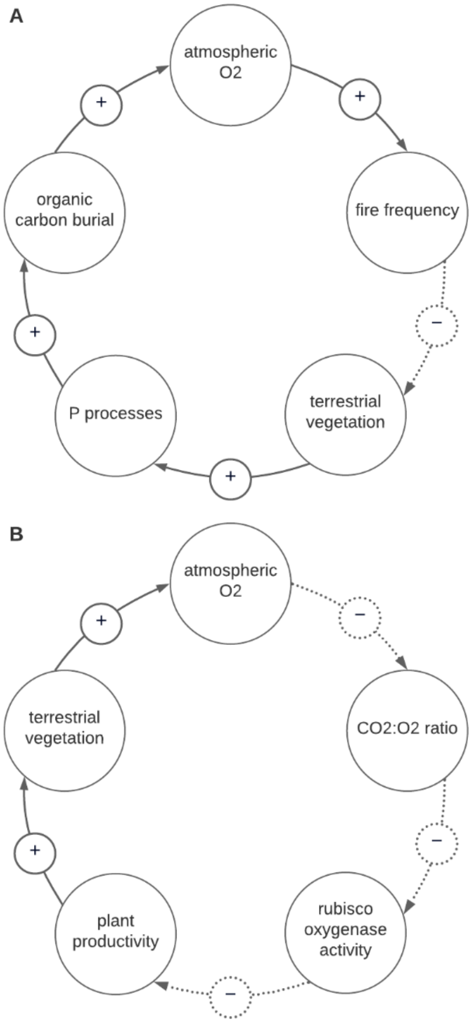

Fig S1. Negative feedbacks on atmospheric oxygen. The sign of the arrows indicates the relationship between the two connecting circles. A solid arrow with a positive sign (+) shows a direct relationship where a change in the first box results in the same change in the second (e.g., increased atmospheric oxygen leads to increased fire frequency). A dashed arrow with a negative sign (-) indicates an inverse relationship in which a change in the first box results in the opposite effect on the second (e.g., increased fire frequency results in decreased terrestrial vegetation). (**A**) fire feedback on atmospheric oxygen. (**B**) photorespiratory feedback on atmospheric oxygen. Here, the box labelled 'P processes' refers to phosphorus redistribution from land to ocean and phosphorus weathering by faster generating vegetation, specific feedbacks are outlined in (1).

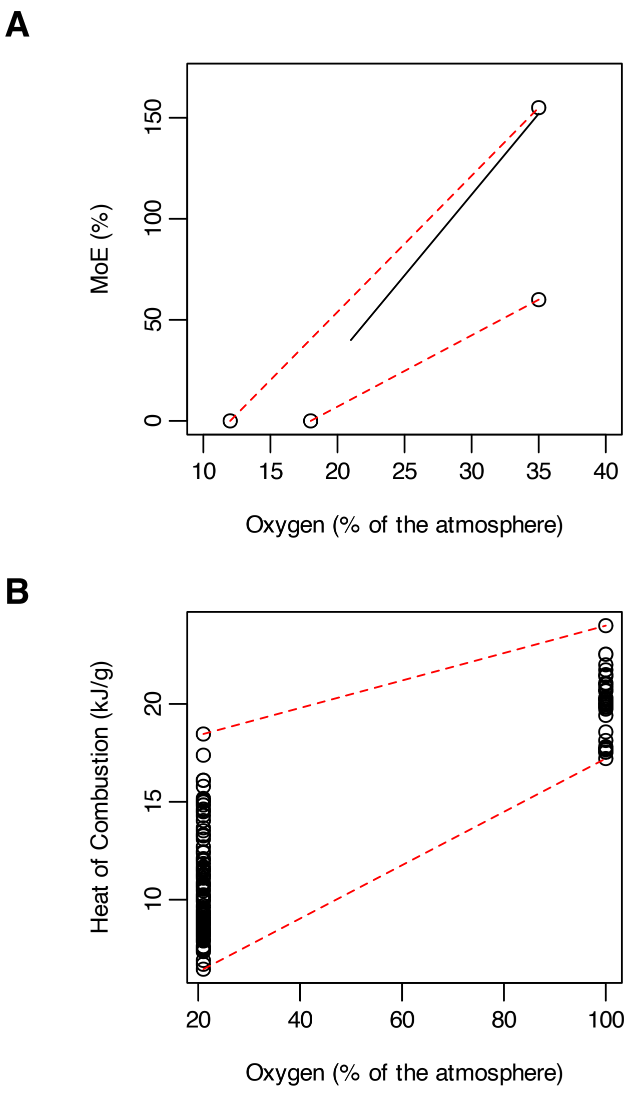

Fig S2. Plots indicating the range used for **A**: Moisture of extinction, where the black solid line indicates the equation defined in Watson and Lovelock (*7*) and **B** Heat of Combustion where circles show data compiled in Vitali et al (*14*). Red dashed lines show the linear minimum and maximum bounds used and outlined in Table S4**.**

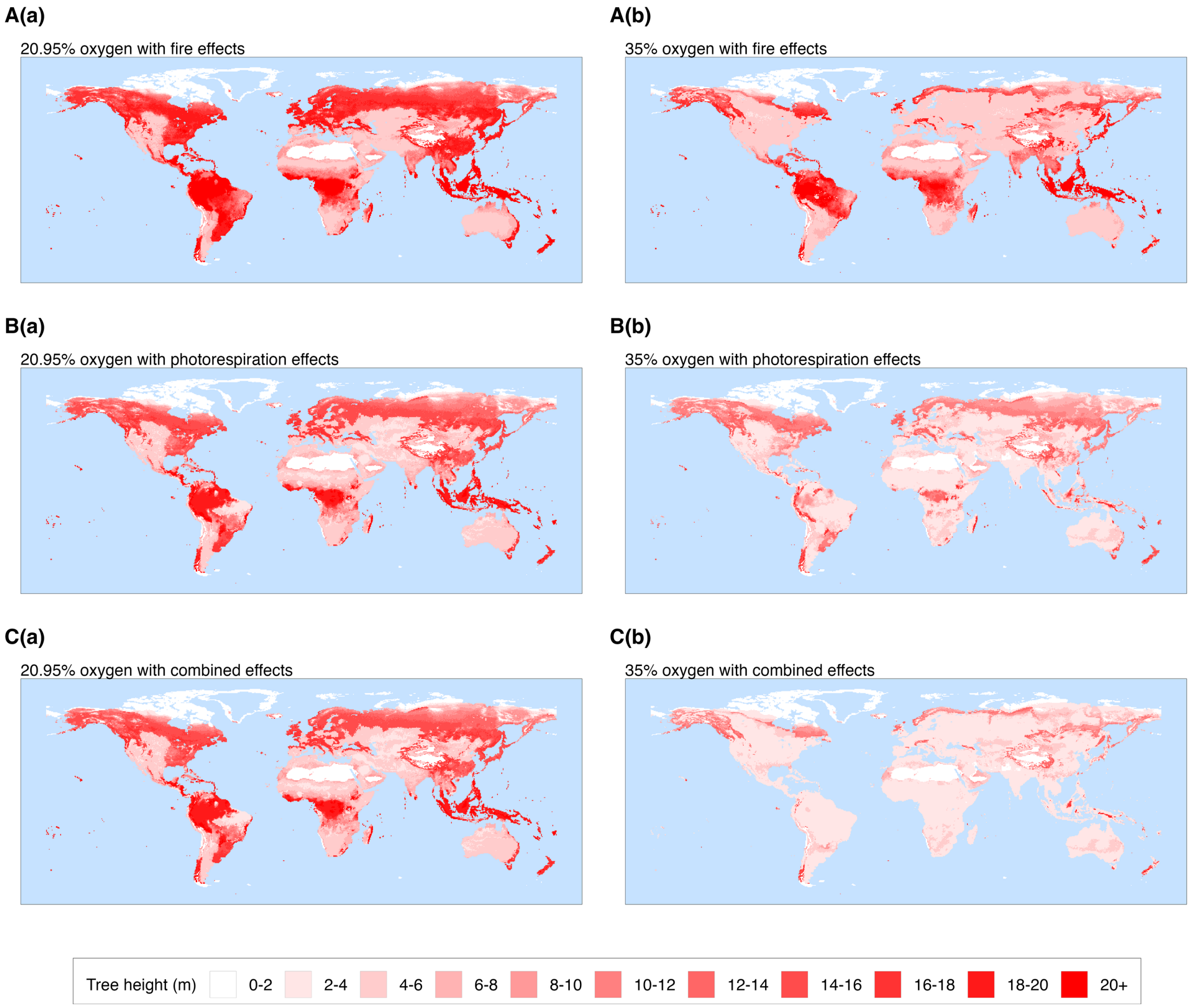

Fig. S3. Tree height over atmospheric oxygen concentrations. (A) oxygen-fire effects only, **(B)** oxygen-photorespiration effects only and **(C)** both oxygen-fire and oxygen-photorespiration effects, and output is plotted for **(a)** 20.95% vol. O_2 (PAL), **(b)** 35% vol. O_2. Plots are taken as 10-year annual averages.

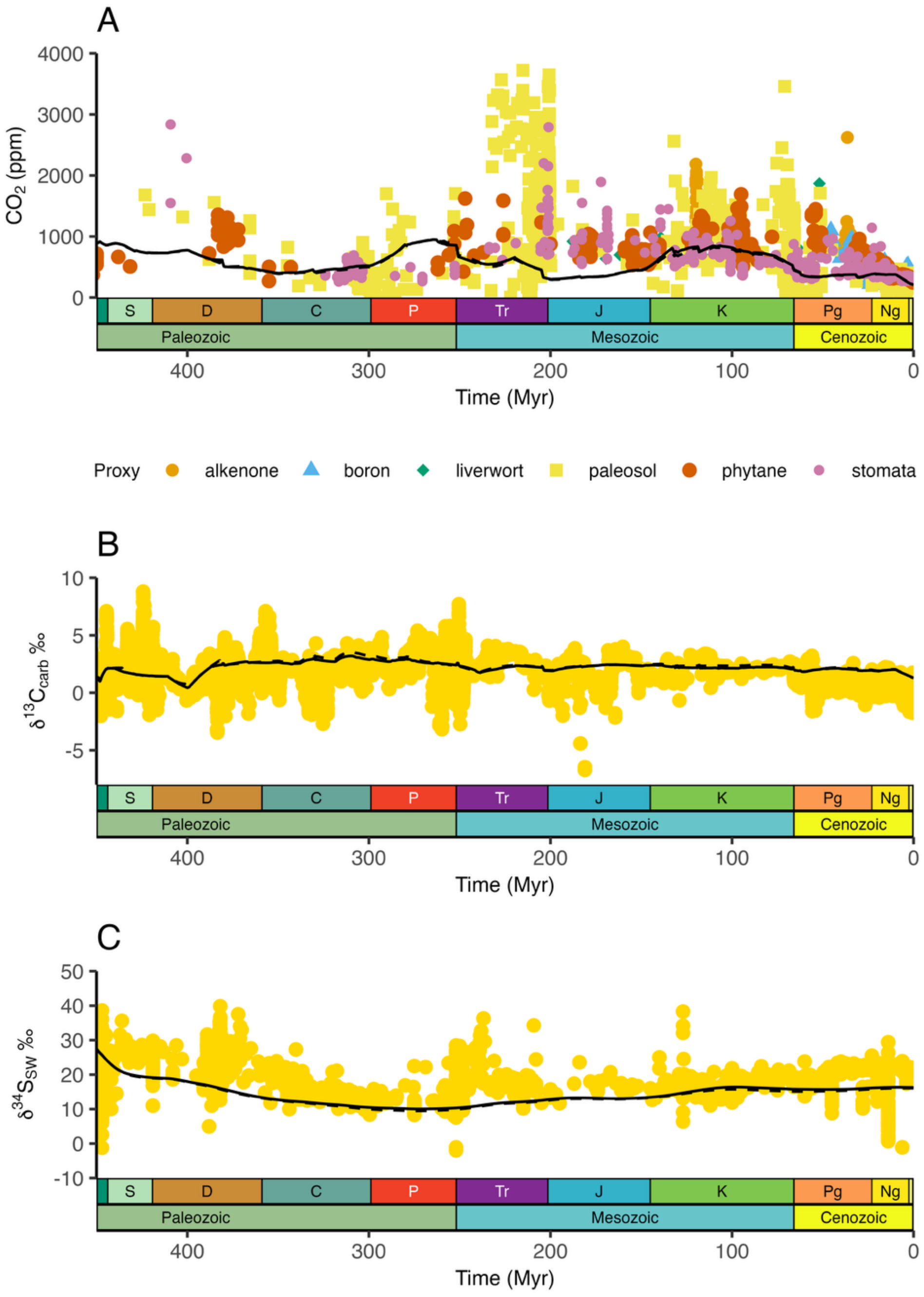

Fig S4. COPSE model comparison to the geological record & proxy data. Model output from standard COPSE configuration (black dashed line) and updated combined fire-photorespiration feedbacks included (solid black line) where both display minor differences from one another. Output is compared against: **A** CO_2 proxy data shown as coloured shapes (*59-60*), **B** geological record of $\delta^{13}C$ (*61*) and **C** geological record in evaporites, barites and CAS (*62*).

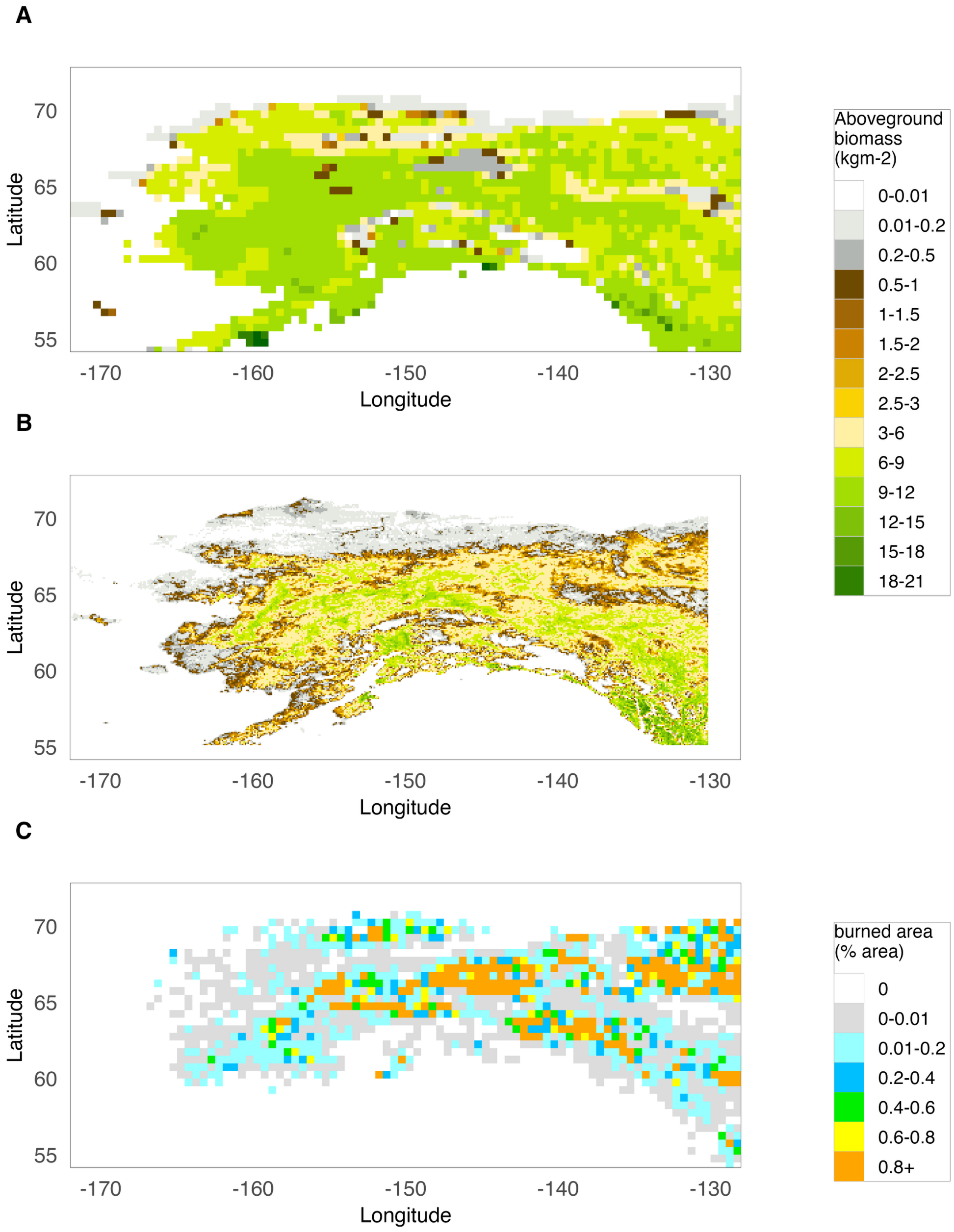

Fig S5. Simulated (A) and observed (B) aboveground biomass and simulated burned area (C) in Alaska. Here simulated aboveground biomass and burned area are plotted as decadal averages from the last 10-years of simulations using the updated version of LPJ-LMfire that includes oxygen effects on fire and photorespiration, with oxygen set to PAL (20.95% vol. O_2). ESA-CCI forest aboveground biomass observational data (*63*) is shown for 2010 and has been aggregated to 10km spatial resolution.

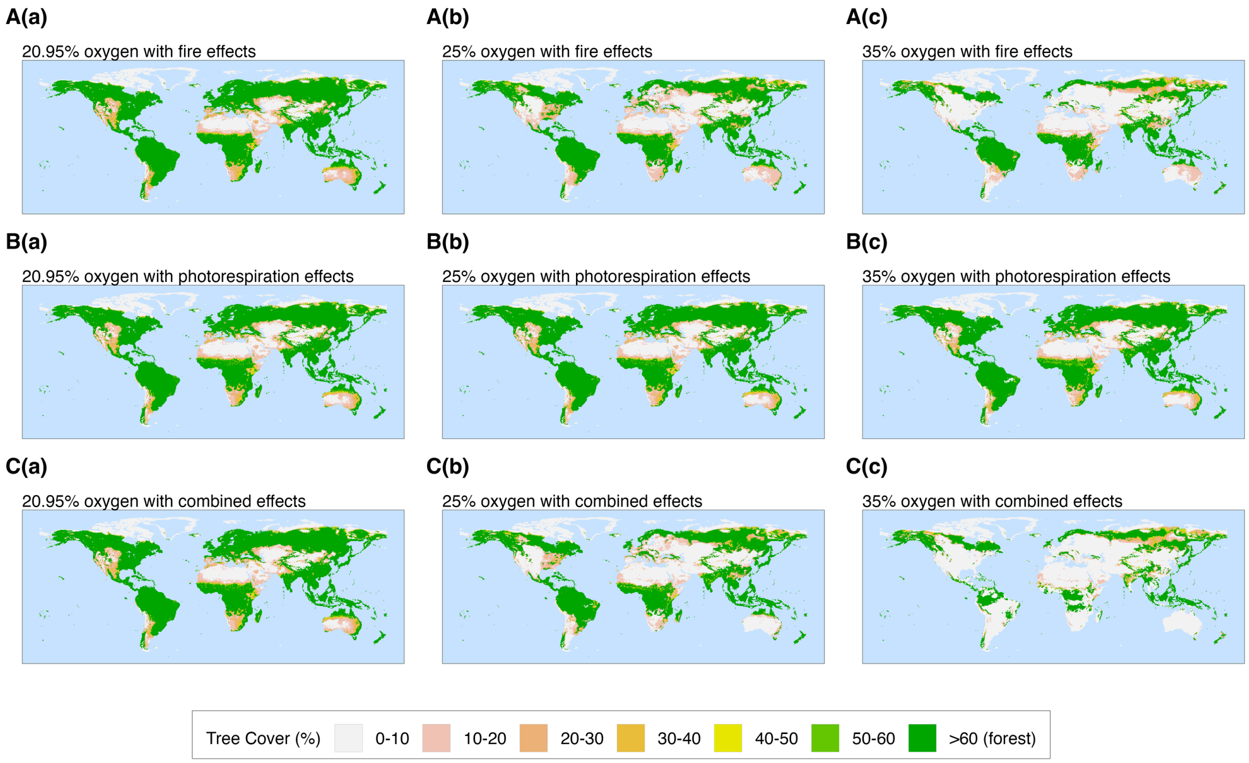

Fig S6. Plots of global tree cover (%) under elevated CO_2. Subplots show **(A)** oxygen-fire effects only, **(B)** oxygen-photorespiration effects only and **(C)** both oxygen-fire and oxygen-photorespiration effects, and output is plotted for **(a)** 20.95% vol. O_2 (PAL), **(b)** 25% vol. O_2 and **(c)** 35% vol. O_2. Plots are taken as 10-year annual averages with forest cover defined to be tree cover greater than 60%.

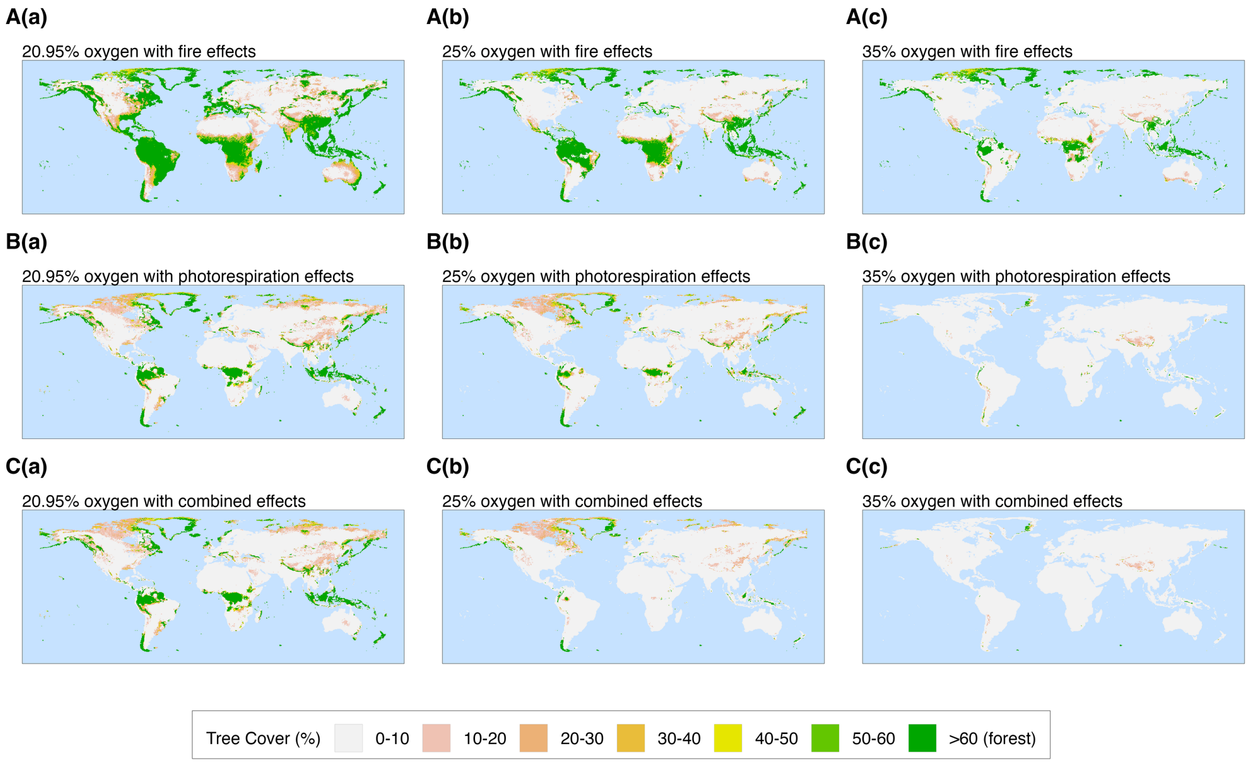

Fig S7. Plots of global tree cover (%) under elevated temperatures. Subplots show **(A)** oxygen-fire effects only, **(B)** oxygen-photorespiration effects only and **(C)** both oxygen-fire and oxygen-photorespiration effects, and output is plotted for **(a)** 20.95% vol. O_2 (PAL), **(b)** 25% vol. O_2 and **(c)** 35% vol. O_2. Plots are taken as 10-year annual averages with forest cover defined to be tree cover greater than 60%. Here elevated temperatures also include a lower meridional temperature gradient, for more details see Methods.

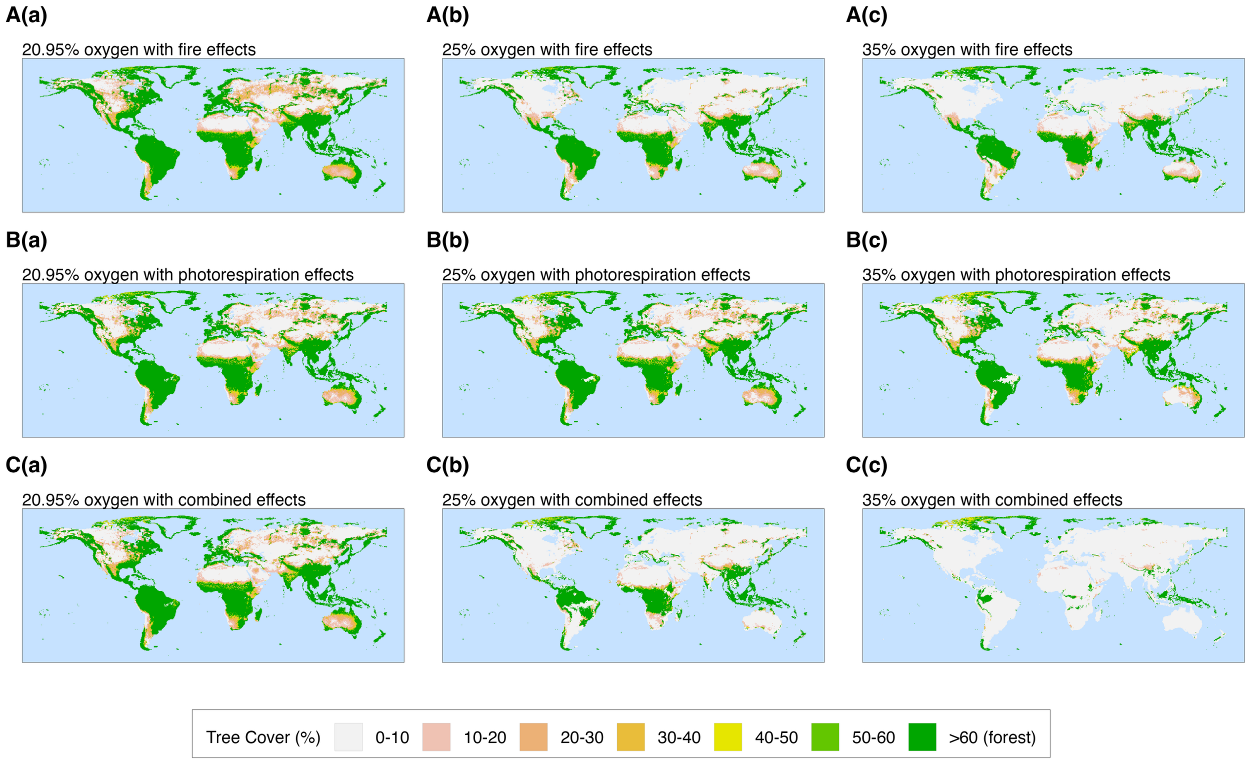

Fig S8. Plots of global tree cover (%) under elevated CO_2 and temperatures. Subplots show **(A)** oxygen-fire effects only, **(B)** oxygen-photorespiration effects only and **(C)** both oxygen-fire and oxygen-photorespiration effects, and output is plotted for **(a)** 20.95% vol. O_2 (PAL), **(b)** 25% vol. O_2 and **(c)** 35% vol. O_2. Plots are taken as 10-year annual averages with forest cover defined to be tree cover greater than 60%. Here elevated temperatures also include a lower meridional temperature gradient, for more details see Methods.

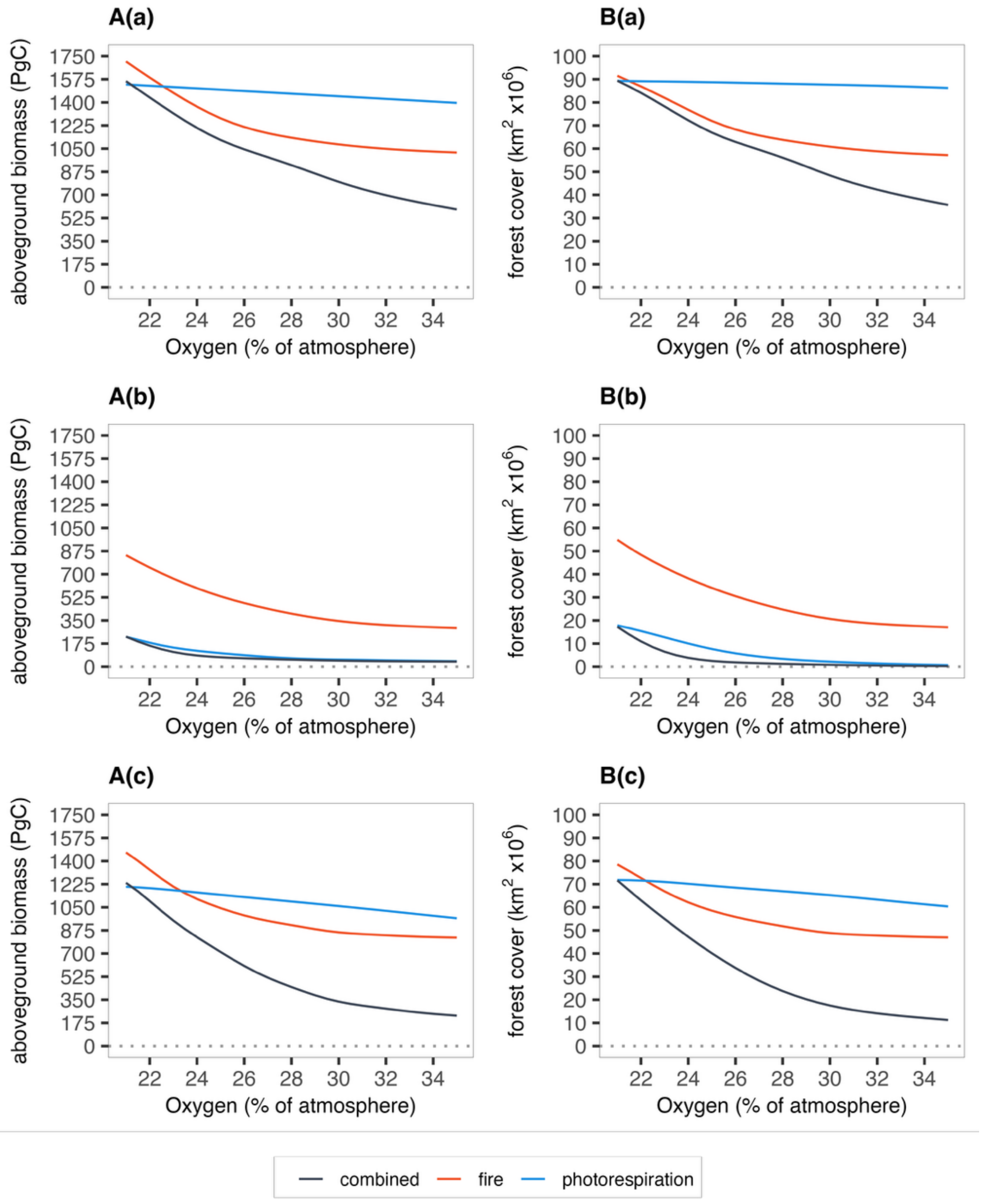

Fig S9. Simulated Global total vegetation over atmospheric oxygen under different climate configurations. Climate configurations include (**a**) elevated CO_2, (**b**) elevated temperature. and (**c**) both elevated CO_2 and temperature, showing **(A)** aboveground biomass (PgC) and **(B)** forest cover (tree cover >60%, km^2). Lines indicate different oxygen simulations which include oxygen-fire effects only (red), oxygen-photosynthesis effects only (blue) and both oxygen-fire and oxygen-photosynthesis effects (black). Totals are calculated from 10-year annual averages from LPJ-LMfire output, summed over grid cells to give a single global value.

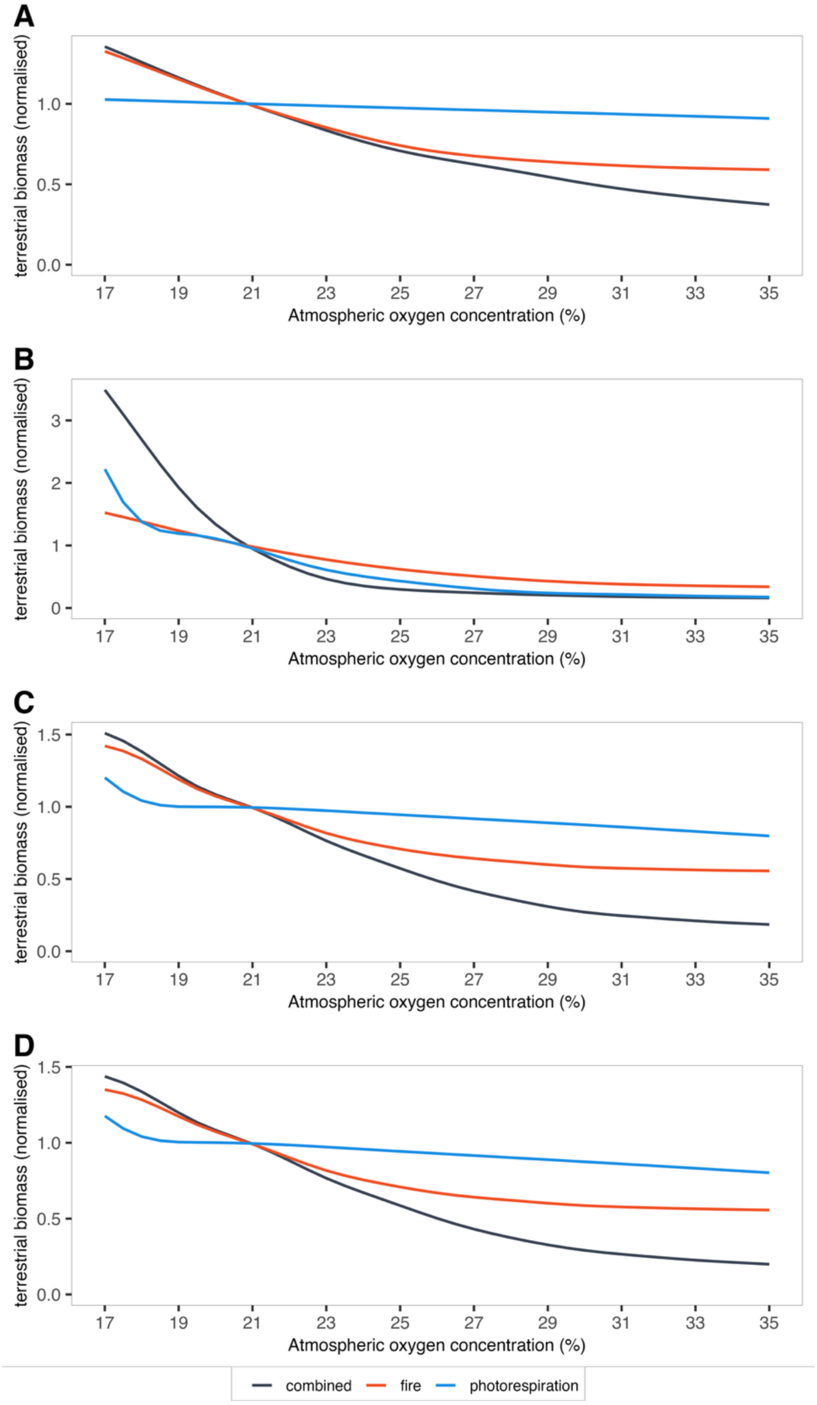

Fig S10. Simulated normalised terrestrial biomass for different climate configurations. Normalized terrestrial biomass as a function of atmospheric oxygen, derived from LPJ-LMfire simulations averaged globally over each oxygen level, used to update scaling factors in COPSE. Lines represent simulations with only oxygen-fire effects (red), only oxygen-photorespiration effects (blue), and combined effects (black). Climate configurations include (**A**) elevated CO_2, (**B**) elevated temperature, (**C**) both elevated CO_2 and temperature and (**D**) elevated CO_2, temperature and precipitation.

Table S1. Reduction (%) of terrestrial biomass and forest cover at 35% vol. O_2 from PAL (20.95 % vol. O_2) for different climate configurations.

Climate configuration	Fire		Photorespiration		Combined	
	biomass	forest cover	biomass	forest cover	biomass	forest cover
Standard	48	46	60	46	86	85
Elevated CO_2	41	38	9	3	63	60
Elevated Temp	66	69	82	96	84	99
Elevated CO_2 & Temp	44	41	20	16	81	84
Elevated CO_2 & Temp & Precip	44	40	20	15	80	83

Table S2. Datasets used to drive simulations in LPJ-LMfire

Variables	Data sets	References
Baseline climatology:<br>Long-term monthly means temperature, precipitation, diurnal temperature range	WorldClim 2.1; Climate WNA	(64-5)
number of days per month with precipitation, wind speed	CRU CL 2.0	(*66*)
total cloud cover	Wisconsin HIRS Cloud Climatology	(*67*)
lightning flashes	LIS/OTD HRMC	(*68*)
Climate interannual variability:<br>Detrended and transient (1871–2010) temperature, precipitation, cloud cover, wind speed, CAPE	20th Century Reanalysis	(*69*)
Elevation and Slope	ETOPO1	(*70*)
Soil particle size distribution and volume fraction of coarse fragments	Harmonized World Soil Database	(*71*)
Atmospheric CO_2 concentrations	Composite CO_2 time series	(*72*)

Table S3. PFT heat of combustion parameters.

	TrBE	TrBR	TeNE	TeBE	TeBS	BoNE	BoS	C3	C4
α_{pft}	-155.03	-144.59	-190.4	-170.85	-185.05	-143.06	-190.02	-134.39	-221.5
β_{pft}	22.14	20.43	22.73	24.34	22.19	21.89	22.17	20.85	19.8

Parameters used to fit equation for each PFT for heat of combustion in the oxygen-abled version of LPJ-LMfire, taken from Vitali et al. (*14*) as outlined in the methods above. With TrBE = tropical broadleaf evergreen, TrBR = tropical broadleaf raingreen, TeNE = temperate needleleaf evergreen, TeBE = temperate broadleaf evergreen, TeBS = temperate broadleaf summergreen, BoNE = boreal needleleaf evergreen, BoS = boreal summergreen, C3gr = C3 perennial grass and C4gr = C4 perennial grass.

Table S4. Overview of values used in the sensitivity simulations

Parameter		Value	Range		Equation	Source
			Min	Max		
Moisture of extinction	MoE	$8O_x$-128	$3.53O_x$-63.53	$6.74O_x$-80.8	8	1,7,11-2
Heat of Combustion	HoC	$\frac{\alpha_{pft}}{O_x} + \beta_{pft}$	$0.14O_x$-3.6	$0.07O_x$-16.99	11	14
Specificity coefficient	τ_{25}	0.132	0.123	0.143	14	18
Rates of night respiration	δ	$\frac{2}{3}$	$\frac{11}{19}$	$\frac{8}{7}$	13	49-50

* HoC is defined for each PFT, see Table S3 for PFT specific values of α_{pft} and β_{pft}

Table S5 Total global aboveground biomass over O_2 from LPJ-LMfire simulations.
Global total values of aboveground terrestrial biomass taken as 10-year annual averages from LPJ-LMfire simulation output. Values are normalized around present atmospheric levels (20.95% vol. O_2) and implemented into the COPSE model as forcing factors for oxygen feedbacks through fire (f_{fire_LPJ}), photorespiration (V_{O2_LPJ}) and the combined feedback including interactions between the two (V_{comb}).

O_2 (%)	Fire		Photorespiration		Combined	
	Total	f_{fire_LPJ}	Total	V_{O2_LPJ}	Total	V_{comb}
20.95	1294.09	1.00	822.82	1.00	837.18	1.00
22	1173.46	0.91	785.47	0.95	642.43	0.77
23	1061.02	0.82	748.83	0.91	489.28	0.58
24	962.59	0.74	712.63	0.87	387.96	0.46
25	889.21	0.69	676.54	0.82	315.05	0.38
26	840.86	0.65	638.78	0.78	261.69	0.31
27	806.24	0.62	601.92	0.73	224.94	0.27
28	777.78	0.60	565.56	0.69	199.01	0.24
29	752.95	0.58	528.63	0.64	177.25	0.21
30	730.57	0.56	492.95	0.60	160.65	0.19
31	712.54	0.55	457.33	0.56	147.96	0.18
32	699.53	0.54	422.12	0.51	137.83	0.16
33	688.75	0.53	388.08	0.47	129.17	0.15
34	680.75	0.53	355.96	0.43	121.85	0.15
35	672.91	0.52	327.43	0.40	116.86	0.14

REFERENCES

1. T. M. Lenton, "Fire feedbacks on atmospheric oxygen," in *Fire Phenomena and the Earth System: An Interdisciplinary Guide to Fire Science*, C. M. Belcher, Ed. (John Wiley & Sons, 2013), pp. 289–308.

2. B. J. W. Mills, A. J. Krause, I. Jarvis, B. D. Cramer, Evolution of atmospheric O_2 through the phanerozoic, revisited. *Annu. Rev. Earth Planet. Sci.* **51**, 253–276 (2023).

3. P. G. Falkowski, The rise of oxygen over the past 205 million years and the evolution of large placental mammals. *Science* **309**, 2202–2204 (2005).

4. T. Lenton, A. J. Watson, *Revolutions that Made the Earth* (Oxford Univ. Press, 2011).

5. L. R. Kump, Terrestrial feedback in atmospheric oxygen regulation by fire and phosphorus. *Nature* **335**, 152–154 (1988).

6. R. A. Berner, D. J. Beerling, R. Dudley, J. M. Robinson, R. A. Wildman Jr., Phanerozoic atmospheric oxygen. *Annu. Rev. Earth Planet. Sci.* **31**, 105–134 (2003).

7. A. J. Watson, J. E. Lovelock, "The dependence of flame spread and probability of ignition on atmospheric oxygen: an experimental investigation," in *Fire phenomena and the Earth system: an interdisciplinary guide to fire science*, C. M. Belcher, Ed. (John Wiley & Sons, 2013), pp. 273–287.

8. L. R. Kump, The rise of atmospheric oxygen. *Nature* **451**, 277–278 (2008).

9. T. M. Lenton, A. J. Watson, Redfield revisited: 2. What regulates the oxygen content of the atmosphere? *Global Biogeochem. Cycles* **14**, 249–268 (2000).

10. A. J. Watson, "Consequences for the Biosphere of Forest and Grassland Fires," thesis, University of Reading (1978).

11. R. A. Wildman, L. J. Hickey, M. B. Dickinson, C. B. Wildman, Burning of forest materials under late Paleozoic high atmospheric oxygen levels. *Geology* **32**, 457–460 (2004).

12. C. M. Belcher, J. M. Yearsley, R. M. Hadden, J. C. McElwain, G. Rein, Baseline intrinsic flammability of Earth's ecosystems estimated from paleoatmospheric oxygen over the past 350 million years. *Proc. Natl. Acad. Sci. U.S.A.* **107**, 22448–22453 (2010).

13. A. Watson, J. E. Lovelock, L. Margulis, Methanogenesis, fires and the regulation of atmospheric oxygen. *Biosystems* **10**, 293–298 (1978).

14. R. Vitali, C. M. Belcher, J. O. Kaplan, A. J. Watson, Increased fire activity under high atmospheric oxygen concentrations is compatible with the presence of forests. *Nat. Commun.* **13**, 7285 (2022).

15. A. U. Igamberdiev, P. J. Lea, Land plants equilibrate O_2 and CO_2 concentrations in the atmosphere. *Photosynth. Res.* **87**, 177–194 (2005).

16. E. G. Nisbet, C. M. R. Fowler, R. E. R. Nisbet, The regulation of the air: A hypothesis. *Solid Earth.* **3**, 87–96 (2012).

17. N. E. Tolbert, C. Benker, E. Beck, The oxygen and carbon dioxide compensation points of C3 plants: Possible role in regulating atmospheric oxygen. *Proc. Natl. Acad. Sci. U.S.A.* **92**, 11230–11233 (1995).

18. M. J. André, Modelling $^{18}O_2$ and $^{16}O_2$ unidirectional fluxes in plants: I. Regulation of pre-industrial atmosphere. *Biosystems* **103**, 239–251 (2011).

19. N. M. Bergman, T. M. Lenton, A. J. Watson, COPSE: A new model of biogeochemical cycling over Phanerozoic time. *Am. J. Sci.* **304**, 397–437 (2004).

20. D. J. Beerling, F. I. Woodward, M. R. Lomas, M. A. Wills, W. P. Quick, P. J. Valdes, The influence of Carboniferous palaeoatmospheres on plant function: An experimental and modelling assessment. *Phil. Trans. R. Soc. Lond. B.* **353**, 131–140 (1998).

21. D. N. Moss, E. G. Krenzer Jr., W. A. Brun, Carbon dioxide compensation points in related plant species. *Science* **164**, 187–188 (1969).

22. M. Pfeiffer, A. Spessa, J. O. Kaplan, A model for global biomass burning in preindustrial time: LPJ-LMfire (v1.0). *Geosci. Model Dev.* **6**, 643–685 (2013).

23. W. J. Bond, F. I. Woodward, G. F. Midgley, The global distribution of ecosystems in a world without fire. *New Phytol.* **165**, 525–538 (2005).

24. I. J. Glasspool, D. Edwards, L. Axe, Charcoal in the Silurian as evidence for the earliest wildfire. *Geology* **32**, 381–383 (2004).

25. R. A. Berner, *The Phanerozoic Carbon Cycle: CO_2 and O_2* (Oxford Univ. Press on Demand, 2004).

26. G. D. Farquhar, S. von Caemmerer, "Modelling of photosynthetic response to environmental conditions," in *Physiological plant ecology II*, O. L. lange, Ed. (Springer, 1982), pp. 549–587.

27. D. B. Jordan, W. L. Ogren, Species variation in the specificity of ribulose biphosphate carboxylase/oxygenase. *Nature* **291**, 513–515 (1981).

28. C. J. Bernacchi, E. L. Singsaas, C. Pimentel, A. R. Portis Jr., S. P. Long, Improved temperature response functions for models of Rubisco-limited photosynthesis. *Plant Cell Environ.* **24**, 253–259 (2001).

29. S.-B. Ku, G. E. Edwards, Oxygen inhibition of photosynthesis. *Planta* **140**, 1–6 (1978).

30. O. Björkman, The effect of oxygen concentration on photosynthesis in higher plants. *Physiol. Plant.* **19**, 618–633 (1966).

31. T. M. Lenton, S. J. Daines, B. J. Mills, COPSE reloaded: An improved model of biogeochemical cycling over Phanerozoic time. *Earth Sci. Rev.* **178**, 1–28 (2018).

32. B. J. Mills, Y. Donnadieu, Y. Goddéris, Spatial continuous integration of Phanerozoic global biogeochemistry and climate. *Gondw. Res.* **100**, 73–86 (2021).

33. J. S. Amthor, The McCree–de Wit–Penning de Vries–Thornley respiration paradigms: 30 years later. *Ann. Bot.* **86**, 1–20 (2000).

34. O. K. Atkin, I. Scheurwater, T. L. Pons, Respiration as a percentage of daily photosynthesis in wholeplants is homeostatic at moderate, but not high, growth temperatures. *New Phytol.* **174**, 367–380 (2007).

35. C. M. Belcher, B. J. W. Mills, R. Vitali, S. J. Baker, T. M. Lenton, A. J. Watson, The rise of angiosperms strengthened fire feedbacks and improved the regulation of atmospheric oxygen. *Nat. Commun.* **12**, 503 (2021).

36. D. C. Wade, N. L. Abraham, A. Farnsworth, P. J. Valdes, F. Bragg, A. T. Archibald, Simulating the climate response to atmospheric oxygen variability in the Phanerozoic: A focus on the Holocene, Cretaceous and Permian. *Clim. Past* **15**, 1463–1483 (2019).

37. J. E. Lovelock, *Gaia: A New Look at Life on Earth* (Oxford Paperbacks, 1979).

38. I. J. Glasspool, A. C. Scott, Phanerozoic concentrations of atmospheric oxygen reconstructed from sedimentary charcoal. *Nat. Geosci.* **3**, 627–630 (2010).

39. C. M. Belcher, M. E. Collinson, A. C. Scott, "A 450-million-year history of fire," in *Fire Phenomena and the Earth System: An Interdisciplinary Guide to Fire Science*, C. M. Belcher, Ed. (Wiley Online Library, 2013), pp. 229–249.

40. D. L. Royer, Y. Donnadieu, J. Park, J. Kowalczyk, Y. Godderis, Error analysis of CO_2 and O_2 estimates from the long-term geochemical model GEOCARBSULF. *Am. J. Sci.* **314**, 1259–1283 (2014).

41. T. P. Jones, W. G. Chaloner, Fossil charcoal, its recognition and palaeoatmospheric significance. *Palaeogeogr. Palaeoclimatol. Palaeoecol.* **97**, 39–50 (1991).

42. A. J. Krause, B. J. W. Mills, S. Zhang, N. J. Planavsky, T. M. Lenton, S. W. Poulton, Stepwise oxygenation of the Paleozoic atmosphere. *Nat. Commun.* **9**, 4081 (2018).

43. R. Tostevin, B. J. Mills, Reconciling proxy records and models of Earth's oxygenation during the Neoproterozoic and Palaeozoic. *Interface Focus* **10**, 20190137 (2020).

44. R. G. Stockey, A. Pohl, A. Ridgwell, S. Finnegan, E. A. Sperling, Decreasing Phanerozoic extinction intensity as a consequence of Earth surface oxygenation and metazoan ecophysiology. *Proc. Natl. Acad. Sci. U.S.A.* **118**, e2101900118 (2021).

45. S. Sitch, B. Smith, I. C. Prentice, A. Arneth, A. Bondeau, W. Cramer, J. O. Kaplan, S. Levis, W. Lucht, M. T. Sykes, K. Thonicke, S. Venevsky, Evaluation of ecosystem dynamics, plant geography and terrestrial carbon cycling in the LPJ dynamic global vegetation model. *Glob. Chang. Biol.* **9**, 161–185 (2003).

46. K. Thonicke, A. Spessa, I. C. Prentice, S. P. Harrison, L. Dong, C. Carmona-Moreno, The influence of vegetation, fire spread and fire behaviour on biomass burning and trace gas emissions: Results from a process-based model. *Biogeosciences* **7**, 1991–2011 (2010).

47. V. Babrauskas, Effective heat of combustion for flaming combustion of conifers. *Can. J. For. Res.* **36**, 659–663 (2006).

48. J. Madrigal, M. Guijarro, C. Hernando, C. Diez, E. Marino, Effective heat of combustion for flaming combustion of Mediterranean forest fuels. *Fire Technol.* **47**, 461–474 (2011).

49. J. de Dios Rivera, G. M. Davies, W. Jahn, Flammability and the heat of combustion of natural fuels: A review. *Combus. Sci. Technol.* **184**, 224–242 (2012).

50. G. J. Collatz, J. T. Ball, C. Grivet, J. A. Berry, Physiological and environmental regulation of stomatal conductance, photosynthesis and transpiration: A model that includes a laminar boundary layer. *Agric. For. Meteorol.* **54**, 107–136 (1991).

51. G. J. Collatz, M. Ribas-Carbo, J. A. Berry, Coupled photosynthesis-stomatal conductance model for leaves of C4 plants. *Funct. Plant Biol.* **19**, 519–538 (1992).

52. A. Haxeltine, I. C. Prentice, BIOME3: An equilibrium terrestrial biosphere model based on ecophysiological constraints, resource availability, and competition among plant functional types. *Global Biogeochem. Cycles* **10**, 693–709 (1996).

53. A. Brooks, G. D. Farquhar, Effect of temperature on the CO_2/O_2 specificity of ribulose-1, 5-bisphosphate carboxylase/oxygenase and the rate of respiration in the light: Estimates from gas-exchange measurements on spinach. *Planta* **165**, 397–406 (1985).

54. E. G. Nisbet, N. V. Grassineau, C. J. Howe, P. I. Abell, M. Regelous, R. E. R. Nisbet, The age of Rubisco: The evolution of oxygenic photosynthesis. *Geobiology* **5**, 311–335 (2007).

55. S. J. Hunter, A. M. Haywood, P. J. Valdes, J. E. Francis, M. J. Pound, Modelling equable climates of the Late Cretaceous: Can new boundary conditions resolve data–model discrepancies? *Palaeogeogr. Palaeoclimatol. Palaeoecol.* **392**, 41–51 (2013).

56. L. Zhang, W. W. Hay, C. Wang, X. Gu, The evolution of latitudinal temperature gradients from the latest Cretaceous through the Present. *Earth Sci. Rev.* **189**, 147–158 (2019).

57. R. A. Berner, D. E. Canfield, A new model for atmospheric oxygen over Phanerozoic time. *Am. J. Sci.* **289**, 333–361 (1989).

58. R. A. Berner, GEOCARB II: A revised model of atmospheric CO_2 over phanerozoic time. *Am. J. Sci.* **294**, 56–91 (1994).

59. G. L. Foster, D. L. Royer, L. Daniel, Future climate forcing potentially without precedent in the last 420 million years. *Nat. Commun.* **8**, 14845 (2017).

60. C. R. Witkowski, J. W. Weijers, S. S. Schouten, Molecular fossils from phytoplankton reveal secular P CO_2 trend over the Phanerozoic. *Sci. Adv.* **4**, aar6091 (2018).

61. M. R. Saltzman, E. Thomas, "Carbon isotope stratigraphy," in *The Geologic Time Scale* (Elsevier, 2012), pp. 207–232.

62. P. W. Crockford, M. Kunzmann, A. Bekker, J. Hayles, H. Bao, G. P. Halverson, Y. Peng, T. H. Bui, G. M. Cox, T. M. Gibson, S. Wörndle, R. Rainbird, A. Lepland, N. L. Swanson-Hysell, S. Master, B. Sreenivas, A. Kuznetsov, V. Krupenik, B. A. Wing, Claypool continued: Extending the isotopic record of sedimentary sulfate. *Chem. Geol.* **513**, 200–225 (2019).

63. M. Santoro, O. Cartus, (2023): ESA Biomass Climate Change Initiative (Biomass_cci): Global datasetsof forest above-ground biomass for the years 2010, 2017, 2018, 2019 and 2020, version 4 (NERC EDS Centre for Environmental Data Analysis, 2023); https://dx.doi.org/10.5285/af60720c1e404a9e9d2c145d2b2ead4e.

64. T. Wang, A. Hamann, D. L. Spittlehouse, T. Q. Murdock, ClimateWNA—High-resolution spatial climate data for western North America. *J. Appl. Meteorol. Climatol.* **51**, 16–29 (2012).

65. R. J. Hijmans, S. E. Cameron, J. L. Parra, P. G. Jones, A. Jarvis, Very high resolution interpolated climate surfaces for global land areas. *Int. J. Climatol.* **25**, 1965–1978 (2005).

66. M. New, D. Lister, M. Hulme, I. Makin, A high-resolution data set of surface climate over global land areas. *Climate Res.* **21**, 1–25 (2002).

67. D. Wylie, D. L. Jackson, W. P. Menzel, J. J. Bates, Trends in global cloud cover in two decades of HIRS observations. *J. Climate* **18**, 3021–3031 (2005).

68. H. J. Christian, R. J. Blakeslee, D. J. Boccippio, W. L. Boeck, D. E. Buechler, K. T. Driscoll, S. J. Goodman, J. M. Hall, W. J. Koshak, D. M. Mach, M. F. Stewart, Global frequency and distribution of lightning as observed from space by the Optical Transient Detector. *J. Geophys. Res. Atmos.* **108**, ACL-4 (2003).

69. G. P. Compo, J. S. Whitaker, P. D. Sardeshmukh, N. Matsui, R. J. Allan, X. Yin, B. E. Gleason, R. S. Vose, G. Rutledge, P. Bessemoulin, The Twentieth Century Reanalysis Project. *Q. J R. Meteorol. Soc.* **137**, 1–28 (2011).

70. C. Amante, B. W. Eakins, *Etopo1 Arc-Minute Global Relief Model: Procedures, Data Sources and Analysis* (US Department of Commerce, National Oceanic and Atmospheric Administration, National Environmental Satellite, Data, and Information Service, National Geophysical Data Center, Marine Geology and Geophysics Division, 2009).

71. N. H. Batjes, *ISRIC-WISE Harmonized Global Soil Profile Dataset* (ISRIC-World Soil Information, 2008).

72. K. M. Krumhardt, J. O. Kaplan, "A spline fit to atmospheric CO2 records from Antarctic ice cores and measured concentrations for the last 25000 years" (ARVE Technical Report 2, ARVE Group, Environmental Engineering Institute, 2012).
